# Biomimetic fusion nanosystem from ginger exosomes and tumor cell membranes: boosting PLK1-targeted therapy in BRCA-heterogeneous HGSOC

**DOI:** 10.1186/s12951-026-04656-z

**Published:** 2026-06-19

**Authors:** Xue Zhou, Yutang Huang, Chenyi Li, Wenlu Mo, Weifeng Xia, Ruiqin Du, Lanxiang Wu, Hongbo Zhao

**Affiliations:** 1 Pharmacogenetics and Pharmacogenomics Laboratory, School of Pharmacy, Chongqing Medical University, Chongqing, 400016 China; 2 Shanghai Key Lab of Reproduction and Development, Shanghai Key Lab of Female Reproductive Endocrine Related Diseases, Obstetrics & Gynecology Hospital of Fudan University, 200433 Shanghai, China

**Keywords:** High-grade serous ovarian cancer, PLK1 inhibitor, Biomimetic nanoplatform, Chronotherapy, Circadian rhythm

## Abstract

High-grade serous ovarian carcinoma (HGSOC) remains a lethal malignancy with few effective therapeutic options. In this study, we systematically evaluated the anti-tumor effect of Bi2536, an inhibitor of Polo-like kinase 1 (PLK1), in HGSOC, and clarified its mechanism. Bi2536 inactivates PLK1, leading to the subsequent inactivation of cyclin-dependent kinase 1 (CDK1). This disruption triggers a cascade of antitumor effects, including G2/M phase arrest, induction of mitochondrial apoptosis, and suppression of cell migration and invasion. Furthermore, we identified circadian oscillations in PLK1 expression both in HGSOC cells and in vivo xenograft models. To enhance therapeutic precision and minimize systemic toxicity, we engineered a biomimetic nano-delivery system for Bi2536. This integrated platform combines chemotherapy and chemodynamic therapy (CDT), significantly improving antitumor outcomes. Importantly, synchronizing Bi2536 administration with the circadian peaks of PLK1 expression further augmented its therapeutic efficacy. In summary, our work establishes that the combination of Bi2536 with a biomimetic nano-delivery system, together with its chronotherapeutic administration, constitutes a highly promising and multifaceted strategy for the treatment of HGSOC.

**Graphical abstract:**

**Figure:**
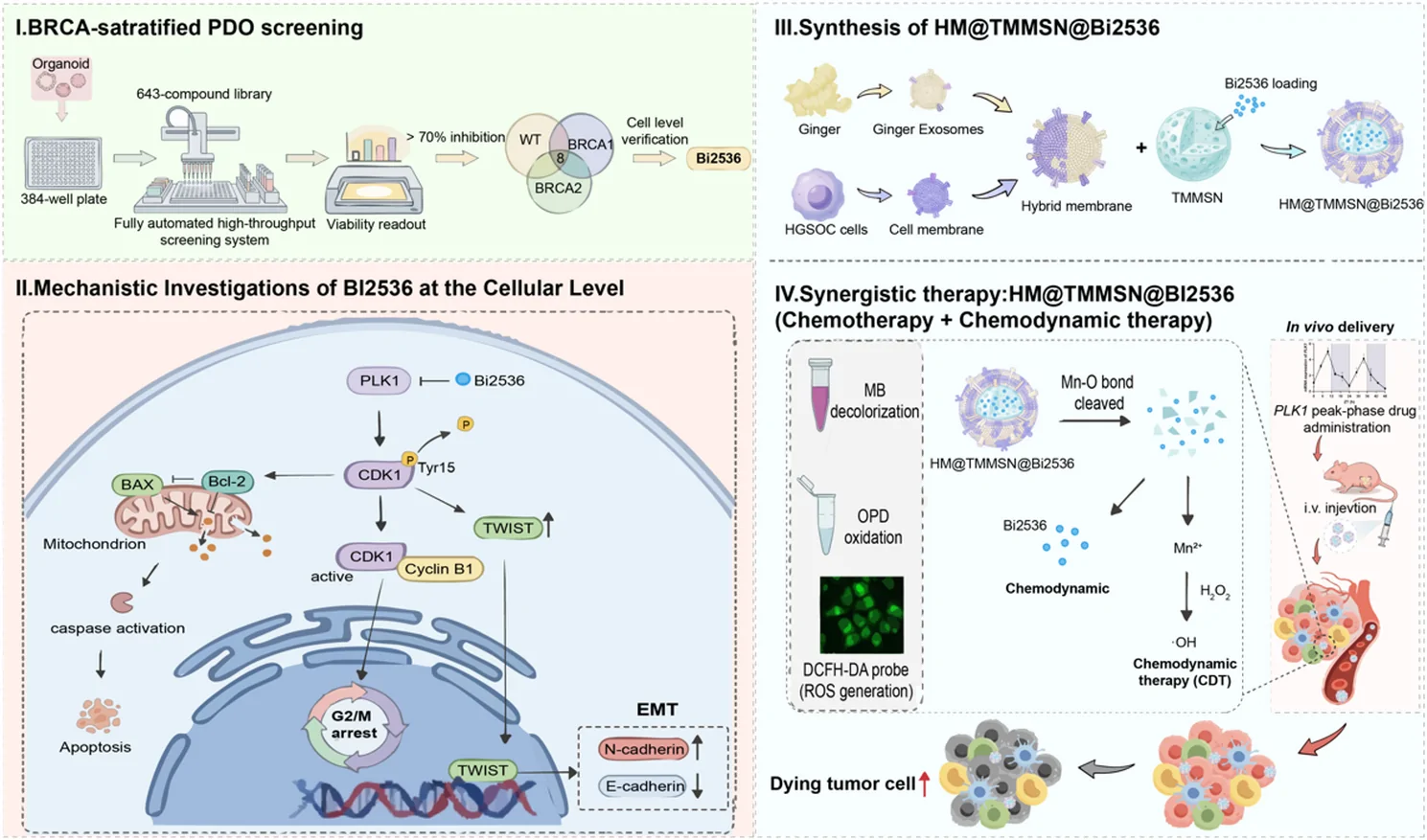

**Supplementary Information:**

The online version contains supplementary material available at https://doi.org/10.1186/s12951-026-04656-z.

## Introduction

HGSOC represents the main histological subtype of epithelial ovarian cancer, and it is still the most deadly gynecological malignant tumor in the world [1–3]. Molecularly, homologous recombination deficiency (HRD) serves as a defining pathogenic feature, with mutations in *Breast Cancer Susceptibility* (*BRCA1/2*) genes constituting its best-characterized subset [4–6]. While poly (ADP-ribose) polymerase (PARP) inhibitors take advantage of the synthetic lethality of *BRCA*-deficient cancers, only a tiny percentage of patients with germline or somatic *BRCA* mutations have a sustained therapeutic response. These restrictions show how vital it is to identify universal treatment objectives that apply to all human genomic backgrounds and create experimental models that accurately capture the great variability of illnesses [7, 8].

Patient-derived organoids (PDOs) have become a powerful platform for drug screening in vitro, which has obvious advantages compared with traditional models. Different from traditional cell lines, PDOs replicate the histological, genetic, and phenotypic characteristics of primary tumors, thus maintaining the heterogeneity among patients required by precision medicine [9, 10]. Bi2536 is a highly selective PLK1 inhibitor that irreversibly inactivates PLK1 through competitive binding to its ATP-binding pocket, leading to sustained G2/M cell cycle arrest [11–13]. Phase II clinical studies of advanced malignant tumors, including non-small cell lung cancer, castration-resistant prostate cancer, and incurable pancreatic cancer, have demonstrated its efficacy [14–17]. However, its further clinical translation is hindered by dose-dependent toxicities, notably neutropenia (occurring in 36% of patients) as well as hepatotoxicity and nephrotoxicity. The therapeutic potential and underlying mechanisms of Bi2536 in HGSOC are still unknown in spite of this data.

The biological clock has a wide range of regulation and control on tumor development and treatment response; It is worth noting that the effectiveness and overall tolerance of chemotherapy can change significantly according to the administration time of a 24-hour cycle, which highlights the key importance of regulating the delivery time of drugs [18–22]. As a core cell cycle kinase embedded within the circadian network, PLK1 itself is predicted to exhibit endogenous circadian rhythms in both its expression and enzymatic activity. Bi2536’s therapeutic index may be destroyed by displacing the peak of drug exposure with the period of highest target vulnerability and minimum host toxicity, since routine and indefinite administration of the drug cannot keep up with this innate biological rhythm. Therefore, developing a chronotherapeutic approach that precisely aligns Bi2536 dosing with the circadian peak of PLK1 activity represents a rational and promising strategy to optimize its antitumor efficacy while mitigating dose-limiting adverse effects.

Plant-derived vesicles (PDVs) have become attractive therapeutic vehicles because of their intrinsic bioactivity, capacity to regulate the immune system, and potential for surface editing [23, 24]. As a natural nano-carrier, PDVs not only retains its inherent anti-tumor characteristics, but also solve the main limitations of artificial drug delivery systems, including secondary biocompatibility and systemic toxicity, and prolong the circulation kinetics [25]. For example, ginger-derived vesicles (GDVs) exhibit dual functionality: they directly suppress tumor cell proliferation in vitro and inhibit xenograft growth in vivo. This underscores their potential not only as stand-alone therapeutics but also as versatile platforms for drug delivery [26]. Despite these advantages, unmodified GDVs exhibit inherent targeting deficiencies that compromise clinical applicability, manifesting as off-target biodistribution, diminished therapeutic efficacy, and dose-limiting adverse effects.

In this work, we confirmed the anti-tumor activity of the PLK1 inhibitor Bi2536 using both in vitro and in vivo models, identifying it as a possible treatment candidate for HGSOC. Through cycle arrest, mitochondrial apoptosis, and inhibition of the epithelial-mesenchymal transition (EMT) pathways, Bi2536 inhibits the progression of HGSOC, according to mechanistic studies. To enhance therapeutic precision, we developed a biomimetic hybrid membrane (HM)-coated, nucleus-targeted manganese-doped mesoporous silica nanoparticle (TMMSN) system for spatiotemporally controlled delivery of Bi2536. By aligning drug administration with circadian rhythm-driven PLK1 expression peaks, we established a “spatio-temporal” therapeutic strategy that maximizes treatment efficacy while minimizing off-target toxicity. This multifunctional platform offers a transformative approach to precision oncology in HGSOC through integrated multimodal synergy.

## Materials and methods

### Subjects and samples

This study was approved by the Ethics Committee Board of the Second Affiliated Hospital of Chongqing Medical University (Approval number: 2024 − 800). HGSOC tissues were obtained from HGSOC patients at Chongqing Medical University’s Second Affiliated Hospital. All participants provided written informed consent.

### Materials

All chemicals were purchased from Sigma-Aldrich or Macklin and used without further purification. Cell Counting Kit-8 (CCK-8), Bi2536, and 4′,6-diamidino-2-phenylindole (DAPI) were purchased from TargetMol. 1,1’-Dioctadecyl-3,3,3’,3’-Tetramethylindodicarbocyanine,4-Chlorobenzenesulfonate Salt (DiD), 3,3’-dioctadecyloxacarbocyanine Perchlorate (DiO), 1,1’-dioctadecyl-3,3,3’,3’-tetramethylindocarbocyanine perchlorate (DiI), Calcein/PI Cell Viability/Cytotoxicity Assay Kit, and 2′,7’-Dichlorodihydrofluorescein diacetate (DCFH-DA) were purchased from Beyotime.

### Establishment and culture of *BRCA*-stratified HGSOC PDOs

PDOs were generated from freshly resected HGSOC specimens obtained from three independent patients per molecular cohort (*BRCA*-wild type, *BRCA1*-mutant, and *BRCA2*-mutant). All organoid lines established were cryopreserved and biobanked (Table S1).

Resected tissues were transported in DMEM/F12 (Gibco) containing 1% penicillin–streptomycin and processed immediately upon arrival. Samples were minced into ~ 2–3 mm³ fragments and subjected to sequential enzymatic dissociation using 0.02% trypsin–EDTA (37 °C, 30 min), followed by collagenase IV digestion of residual tissue (37 °C, 30 min) with intermittent trituration. After filtering, the suspension was centrifuged for 5 min at 400 g. Cell pellets were resuspended and embedded at a minimum density of 2 × 10⁴ cells per dome in growth factor-reduced Matrigel domes (25–50 µL per dome; Corning). After polymerization, domes were overlaid with 300 µL of tumor organoid medium (TOM; DMEM/F12-based) supplemented with N2, B27, Primocin, epidermal growth factor (EGF), fibroblast growth factor 2 (FGF‑2), hepatocyte growth factor (HGF), N‑acetyl‑L‑cysteine, L‑glutamine, CHIR99021, R‑spondin 1, A83‑01, prostaglandin E2 (PGE2), and Y‑27,632.

Organoids were maintained at 37 °C in 5% CO₂ with medium changes every 2–3 days. For routine maintenance, organoids were passaged at a 1:2 ratio every 7–10 days upon reaching 200–300 μm in diameter using mechanical shearing or Accutase-based dissociation.

### High-throughput compound screening

About 500 PDOs per well were obtained by resuspending mature PDOs in an ideal organoid medium and distributing them into 384-well plates at a volume of 32 µL per well. A library of 643 small-molecule compounds was added to the plates via a high-throughput automated instrument (ExplorerTM G3), adjusting the final compound concentration to 10 µM. After 48 h of co-incubation at 37 °C with 5% CO₂, cell viability was assessed using the CCK-8 assay. Dimethyl sulfoxide (DMSO, 0.1% v/v) and paclitaxel (10 nM) served as negative and positive controls, respectively. Each compound was tested in one well per screening round. For each *BRCA*-defined PDO cohort, the entire screening was repeated in three independent runs on separate occasions to ensure reproducibility.

### Histological and immunofluorescence characterization

For histological evaluation, organoids were released from Matrigel, fixed in 4% paraformaldehyde, and embedded in 2% agarose to generate cell blocks. Blocks were processed for paraffin embedding using standard dehydration and clearing steps, and sections were cut at 4–5 μm for hematoxylin and eosin (H&E) staining.

Lineage fidelity was assessed by immunofluorescence staining of tumor protein 53 (p53) and Cytokeratin 7 (CK7) on paraffin sections. After deparaffinization and antigen retrieval, sections were blocked with 10% goat serum and incubated with primary antibodies overnight at 4 °C, followed by fluorophore-conjugated secondary antibodies and DAPI nuclear counterstaining. Whole-slide images were acquired using a Pannoramic MIDI II fluorescence scanner (3D Histech).

### Cell culture and reagents

The HGSOC cell lines OVCAR3, UWB1.289, and KURAMOCHI were obtained from the Cell Bank of the Chinese Academy of Sciences (Beijing, China) and Procell Life Science & Technology Co., Ltd. (Wuhan, China). All cell lines were routinely authenticated by short tandem repeat (STR) profiling.

OVCAR3 cells were cultured in RPMI-1640 complete medium supplemented with 20% fetal bovine serum (FBS) and insulin as recommended by the American Type Culture Collection. UWB1.289 cells were maintained in complete growth medium composed of 50% RPMI-1640 and 50% MEGM (Lonza MEGM BulletKit, CC-3150) supplemented with 3% FBS. KURAMOCHI cells were maintained in RPMI-1640 complete medium with 20% FBS. All cultures were maintained at 37 °C in a humidified incubator with 5% CO_2_.

To create a stock solution, Bi2536 was dissolved in DMSO. Just before usage, the stock solution was diluted in the full culture medium. For all treatment groups, the final DMSO concentration was kept constant at ≤ 0.1%, v/v.

### In vitro cell phenotypic assays

96-well plates were seeded with 5 × 10^3^ cells for varying times in order to perform the CCK-8 test. CCK-8 was then used to assess cell viability. 0.8 × 10^3^ cells were cultivated for two weeks after being seeded in 6-well plates for the colony formation assay. Following methanol fixation, crystal violet staining was applied to the cells, and cell colonies greater than 0.1 mm in diameter were counted. The experiments for cell migration and invasion involved seeding 0.5 × 10^5^ cells into the upper compartments of “Transwell” chambers (BD Biosciences, Germany) that were either precoated or not with Matrigel. The bottom chamber was then injected with a 20% serum solution. The cells on the bottom were fixed with 4% paraformaldehyde, stained with 0.5% crystal violet, and counted following a 48 h incubation period.

To assess cell apoptosis and cell cycle, cultured cells were digested with trypsin, washed with phosphate-buffered saline (PBS), and stained with annexin V-fluorescein isothiocyanate and propidium iodide (PI). Cell apoptosis rate and cell cycle were analyzed using a CytoFLEX flow cytometer (Beckman Coulter, CA, USA).

### Protein isolation and Western blotting analysis

The Pierce bicinchoninic acid (BCA) protein assay kit (Thermo Fisher Scientific, USA) was used to extract and quantify the total protein. It was then separated using 10% SDS-PAGE and transferred to polyvinylidene fluoride (PVDF) membranes. After blocked with 5% BSA for 1 h, the membrane was incubated at 4 °C overnight with primary antibodies as follows: PLK1 [1:5,000, Immunoway, Cat# YM8488), p-PLK1(T210) [1:1,000, Immunoway, Cat# YP0964], CyclinB1 [1:1,000, Immunoway, Cat# YT1169], p-CDK1 [1:2,000, ABclonal, Cat# AP1350], cleaved-caspase-3 [1:1,000, ABclonal, Cat# A11021], cleaved-PARP [1:20,000, ABclonal, Cat# A19612], BCL-2 (1:5,000, Proteintech, Cat# 12789-1-AP), BAX [1:50,000, Proteintech, Cat# 50599-2-lg], TWIST [1:1,000, ABclonal, Cat# A25134], N-cadherin [1:40,000, Proteintech, Cat# 66219-1-Ig], E-cadherin [1:1,500, Proteintech, Cat# 60335-1-Ig], and GAPDH (1:5,000, Bioss, Cat# bsm-33033 M). Following incubation with horseradish peroxidase (HRP)-conjugated secondary antibodies, immunoreactive bands were detected by ECL Prime (GE Healthcare, UK) and an LAS-3000 imager (Fujifilm, Japan).

### Circadian rhythm induction

In order to evaluate the rhythm oscillation, the synchronous cells were synchronized with dexamethasone (100 nM) for 2 h, then the cells were washed with PBS and replaced with a new medium. This time point is defined as zeitgeber time 0 (ZT0). Total RNA was collected every 4 h for 64 h (≥ 2 circadian cycles). At each time point, cells were lysed immediately for RNA extraction to minimize RNA degradation.

### Real-time quantitative polymerase chain reaction (RT-qPCR)

TRIzol reagent (Invitrogen, USA) was used to extract total RNA in accordance with the manufacturer’s instructions. cDNA was synthesized using PrimeScript™ RT reagent Kit with gDNA Eraser (Takara, Japan).

SYBR Green master mix (Accurate Biology, China) was used for RT-qPCR on a Bio-Rad CFX Connect system (Bio-Rad, USA). *GAPDH* served as the internal reference control for quantitative PCR (qPCR) amplification to measure *PLK1* mRNA expression levels, following the manufacturer’s protocol. Information on primer sequences is shown in Table S2. The 2^−ΔΔCt^ method was used to calculate fold changes in gene expression normalized to the control.

### Preparation and identification of HM

GDVs and HGSOC cell membrane (HCM) were separated by differential ultracentrifugation and a membrane extraction kit (Beyotime), respectively [27, 28]. The bicinchoninic acid (BCA) test was used to measure the protein concentration of the purified HCM and GDVs. To create the biomimetic HM, HCM and GDVs were then combined at a 1:1 mass ratio, sonicated, and then successively extruded through 400 nm and 200 nm polycarbonate membranes.

To confirm successful membrane fusion, the protein profiles of HCM and GDVs were analyzed by SDS-PAGE with Coomassie Brilliant Blue staining. Furthermore, HCM was labeled with a fluorescence resonance energy transfer (FRET) donor-acceptor pair (DiI and DiD probes) and incubated with increasing amounts of GDVs. Fluorescence emission spectra were recorded between 550 and 750 nm on a fluorescence spectrometer (Edinburgh FS5, UK) with an excitation wavelength of 525 nm and a slit width of 3 nm.

### Synthesis and characterization of HM@TMMSN@Bi2536

A mixture of 6 mL ethanol and 60 mL H_2_O was used to dissolve 660 µL triethanolamine (TEA) and cetyltrimethylammonium bromide (CTAB, 1.20 g). Then, while stirring, 6 mL of MnSO_4_ (8 g/L) was added drop by drop. Tetraethyl orthosilicate (TEOS, 0.90 mL) and (3-Aminopropyl) triethoxysilane (APTES, 0.15 mL) were then gradually added dropwise to the solution after it had been heated to 80 °C. After reacting for 4 h, the brown product was collected, and the CTAB surfactant was extracted using hot NaCl−methanol (8 g/L) under reflux. Finally, the product was lyophilized for 24 h to obtain MMSN.

MMSN were covalently conjugated with the nuclear-targeting HIV-1 Trans-Activator of Transcription (TAT) peptide via the 1-Ethyl-3(3-(dimethylamino)-propyl)carbodiimide (EDC) and N-hydroxysuccinimide (NHS) mediated carbodiimide coupling reaction. Briefly, MMSN were dispersed in 0.05 mol/L 2-Morpholinoethanesulphonic acid (MES) buffer by sonication for 30 min. After dissolving the TAT peptide to a concentration of 2 g/L in the same MES buffer, EDC and NHS were added at a molar ratio of EDC: TAT: NHS = 10:1:2. The mixture was magnetically stirred at room temperature for 30 min in the dark to activate the carboxyl groups of TAT. Following a dropwise addition of the activated TAT solution to the MMSN suspension (TAT: MMSN mass ratio = 1:10), the mixture was gently shaken and allowed to react for 24 h at 4 °C in the absence of light. The resulting TAT-conjugated MMSN (TMMSN) were purified by repeated centrifugal washing (4000 g, 10 min per cycle) with PBS buffer (pH 7.4) using an ultrafiltration centrifugal tube (MWCO = 100 kDa) to remove unreacted peptide and excess reagents.

Drug solutions with different concentrations were prepared, and their absorbance values were measured to construct a standard curve. To prepare TMMSN@Bi2536, Bi2536 was mixed with TMMSN at various mass ratios, shaken on a shaker for 24 h, and then centrifuged at 15,000 g for 30 min. The resultant precipitate was gathered, and the drug loading efficiency (LE) and encapsulation efficiency (EE) were computed using the absorbance value of the supernatant. The collected precipitate was washed with ultrapure water three times and finally lyophilized for 24 h to obtain TMMSN@Bi2536. Subsequently, TMMSN@Bi2536 was added to HM, and the mixture was extruded through 400 nm and 200 nm polycarbonate membrane filters sequentially to form HM@TMMSN@Bi2536.

The size and zeta potential were measured by a dynamic light scattering detector (Zetasizer, Malvern, UK). The morphology was visualized using transmission electron microscopy (Hitachi TEM system, Japan) as previously described. Elemental composition analysis was performed utilizing X-ray Photoelectron Spectroscopy (XPS, Thermo Scientific K-Alpha, USA).

### Drug and Mn releasing performance

In the in vitro drug release experiment, 10 mg of HM@TMMSN@Bi2536 was dispersed in 25 mL of PBS with different pH levels (7.4 or 5.5), then shaken at 200 g and 37 °C in the dark. At regular intervals, 2 mL was removed and replaced with the same volume of fresh PBS. The samples were centrifuged (15,000 g for 30 min) to calculate Bi2536 release by measuring absorbance.

Mn Release from Nanoparticles 10 mg of HM@TMMSN@Bi2536 were dispersed in PBS (20 mL, pH = 5.5 or 7.4) under magnetic stirring. An aliquot (0.5 mL) was extracted from the solution at different time intervals (0, 2, 4, 8, 10, 12, 24, 36, 48) and centrifuged (13000 rpm, 15 min) to remove remaining particles. The supernatant was obtained for detecting the concentration of released Fe element by Inductively Coupled Plasma Mass Spectrometry (ICP-OES).

### Extracellular reactive oxygen species (ROS) detection

Methylene Blue (MB) was used to detect hydroxyl radicals (•OH) [29]. The reaction system with different concentrations of HM@TMMSN was incubated with hydrogen peroxide (H_2_O_2_, 100 µM) and MB(10 mg/L), and the reaction mixture was gently shaken in the dark for 30 min at 37 °C.

At the same time, o-phenylenediamine (OPD) test was used to further evaluate ROS production. This test relies on oxidative polymerization of ROS in OPD to form yellow 2,3- diaminopropionic acid (DAP) with a typical absorption peak of 450 nm [30]. Briefly, HM@TMMSN was dispersed in PBS buffer (pH 6.5) to prepare suspensions with different concentrations. Subsequently, the HM@TMMSN suspension was mixed with OPD solution (final concentration: 0.1 mM) and H_2_O_2_ (final concentration: 100 µM). For 30 min, the mixture was incubated in the dark at 37 °C. A microplate reader was used to measure the absorbance spectra following incubation.

### Intracellular ROS detection

DCFH-DA method was used to determine the production of ROS in cells. Briefly, OVCAR3, UWB1.289, and KURAMOCHI cells were inoculated in a 24-well plate and cultured for 24 h to allow cell adhesion. The adhered cells were coincubated with HM@TMMSN (30 mg/L) for 4 h, then washed with PBS and incubated with culture medium containing DCFH-DA for 30 min. Intracellular ROS was immediately detected and photographed by inverted fluorescence microscopy.

### Live/dead staining for in vitro cytotoxicity

Cells were treated with PBS (control), free Bi2536, HM@TMMSN, or HM@TMMSN@Bi2536 under the indicated conditions. After incubation for 4 h, cells were stained with calcein-AM and PI for 30 min in the dark (working concentrations per kit instructions) and imaged by fluorescence microscopy. Green (calcein-AM) and red (PI) signals were used to visualize viable and membrane-compromised cells, respectively.

### Cellular uptake

OVCAR3, UWB1.289, and KURAMOCHI cells were seeded in a 24-well plate and incubated for 24 h, then the medium was replaced with medium containing HM@TMMSN, while the control was cultured in fresh medium alone. Following the removal of the media after four hours, the cells underwent PBS washes, a fixation with 4% paraformaldehyde (PFA) for 20 min, additional PBS washes, DAPI staining for 6 min, and PBS washes. Confocal laser scanning microscopy (CLSM) was used to image cells.

### Hemolysis experiment

Briefly, fresh mouse blood was centrifuged to collect red blood cells (RBCs), which were then washed three times with PBS buffer (pH 7.4) and resuspended to a 2% (v/v) RBC suspension. Different concentrations of HM@TMMSN and HM@TMMSN@Bi2536 were mixed with the 2% RBC suspension, respectively. PBS buffer and deionized water served as the negative control (0% hemolysis) and positive control (100% hemolysis), respectively. After one hour of incubation at 37 °C, each combination was centrifuged for 5 min at 3000 g. The absorbance of the supernatant at 541 nm was measured using a microplate reader. The hemolysis rate was calculated as follows: Hemolysis rate (%) = [(A_sample_-A_negative_)/(A_positive_-A_negative_)] × 100%. A hemolysis rate below 5% was considered biocompatible.

### Cell-derived xenograft (CDX) model

The Chongqing Medical University Animal Ethics and Experimental Committee gave its approval to all animal experiments, which were conducted in compliance with the National Institutes of Health’s Guide for the Care and Use of Laboratory Animals. Female BALB/c-nude mice weighing 16–20 g and aged 4–6 weeks were kept in a particular pathogen-free environment at Chongqing Medical University’s Department of Laboratory Animal Science. In order to create the subcutaneous xenograft tumor model, 3 × 10^6^ HGSOC cells were implanted subcutaneously into mice. Mice were randomly assigned to various experimental groups once the tumor volume reached 150 mm^3^, and they received weekly intratumoral injections of PBS, Bi2536 (25 mg/kg, 50 mg/kg), and HM@TMMSN@Bi2536 (94.3 mg/kg) for three weeks. The HGSOC cell line was injected into the mice’s peritoneal cavity at a density of 3 × 10^6^ cells per mouse in order to simulate intraperitoneal metastasis. The subcutaneous or metastatic tumors were removed for additional study once the experiments were over and the animals were euthanized.

### LC‑MS/MS analysis

Chromatographic separation was performed on an Agilent 1290 Infinity II series ultra‑high‑performance liquid chromatography system (Agilent Technologies, Santa Clara, CA, USA) using a Waters ACQUITY UPLC BEH C18 column (2.1 mm × 100 mm, 1.7 μm; Waters, Milford, MA, USA) maintained at 35 °C. The mobile phase consisted of 0.1% formic acid in water (phase A) and methanol (phase B). The flow rate was set to 300 µL min⁻¹, the injection volume was 2 µL, and the autosampler temperature was maintained at 10 °C.

Mass spectrometric detection was conducted on an Agilent 6495 triple quadrupole mass spectrometer equipped with an Agilent Jet Stream electrospray ionisation source (Agilent Technologies) operated in positive ion mode. The ion source parameters were as follows: capillary voltage, + 3000 V; nozzle voltage, + 1500 V; drying gas (N₂) temperature, 250 °C; drying gas flow, 11 L min⁻¹; sheath gas (N₂) temperature, 400 °C; sheath gas flow, 12 L min⁻¹; and nebuliser pressure, 35 psi.

### Calibration curve

The calibration curve was constructed by plotting the peak area of BI‑2536 against the corresponding concentration. Least‑squares linear regression with a weighting factor of 1/x was applied. The regression equation was y = 4,384,290.386101x + 16,5841.625896, where y represents the peak area and x represents the concentration in mg L⁻¹. The coefficient of determination (R²) exceeded 0.999. Calibration points were retained only when the signal‑to‑noise ratio was ≥ 10, the measured recovery fell within 80–120%, and the final calibration curve included at least five of the eight prepared standard concentrations.

### Statistical analysis

To conduct statistical studies, GraphPad Prism (version 8.0) was used. The mean ± standard deviation (SD) is used to display the results. The two treatment groups were compared using the unpaired Student’s t-test (two-tailed). A paired t-test was used for groups that were paired. Three or more groups were contrasted using ANOVA. *P* < 0.05 was regarded as a statistically significant confidence level.

## Results

### Establishment of HGSOC-PDO platform and identification of Bi2536 as a potent inhibitor

**Fig. 1:**
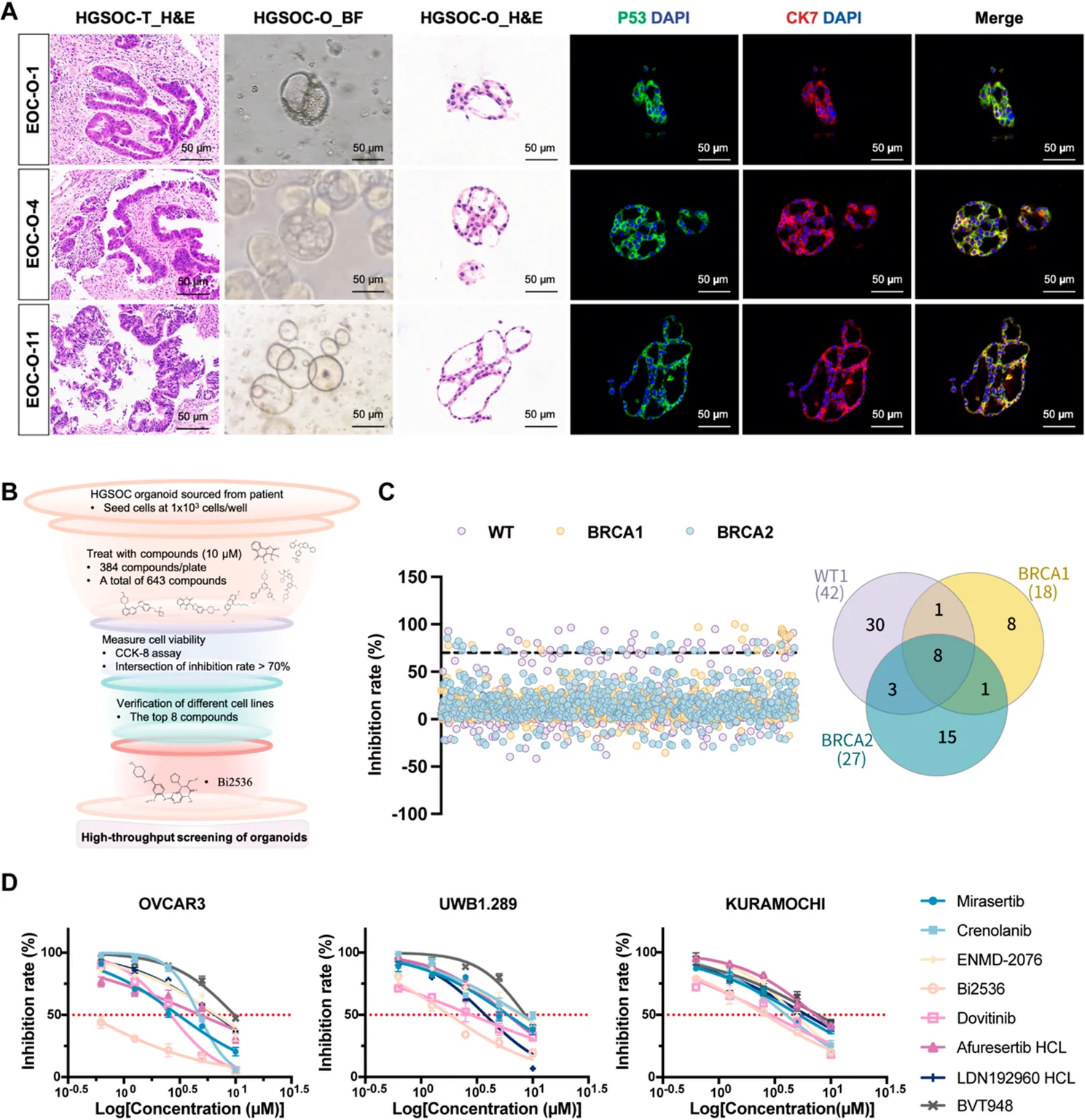
Establishment of *BRCA*-stratified HGSOC PDO screening platform and identification of Bi2536 as a lead compound. (**A**) Validation of PDO fidelity to primary HGSOC tissues. Shown are representative H&E staining of tumors and matched PDOs, bright-field PDO morphology, and immunofluorescence for p53 and CK7 (DAPI counterstain; scale bars: 50 μm). (**B**) Schematic overview of the *BRCA*-stratified HGSOC PDO workflow for high-throughput compound screening and hit selection. (**C**) High-throughput screening results across *BRCA*-stratified PDO cohorts (three independent runs per cohort). A Venn diagram highlights overlapping hits meeting the predefined inhibition threshold (> 70% growth inhibition) in all three *BRCA* genotypes. (**D**) Secondary validation of candidate compounds in HGSOC cell lines (OVCAR3, UWB1.289, KURAMOCHI)

To identify novel therapeutic candidates for HGSOC, we established a PDO screening platform that recapitulates critical pathological and molecular features of primary tumors. Fresh HGSOC specimens were enzymatically dissociated, embedded in Matrigel, and cultured under three-dimensional conditions to propagate tumor-derived populations [31]. Histopathological analysis confirmed that the resulting PDOs faithfully preserved original tumor phenotypes, exhibiting high-grade nuclear atypia, a hallmark feature of HGSOC, in both primary tumors and corresponding PDOs. Immunohistochemical staining further demonstrated sustained strong expression of the epithelial marker CK7, alongside persistent aberrant p53 immunoreactivity (Fig. 1A, S1) [4, 32].

We subsequently conducted high-throughput screening using an expanded panel of PDOs categorized by *BRCA* mutation status, including wild-type (WT), *BRCA1*-mutant, and *BRCA2*-mutant lines. A library of 643 small-molecule compounds was screened at a fixed concentration of 10 µM, with organoid viability suppression as the primary efficacy readout. Compounds achieving > 70% growth inhibition across all three *BRCA* genotypes were selected through intersection analysis, yielding eight pan-subtype inhibitors (Fig. 1B-C, Fig. S2). In order to determine the most promising candidate drugs, eight drugs were observed in HGSOC cell lines with different genetic characteristics in theory of three Represents: OVCAR3 (wild type *BRCA*),UWB1.289 (*BRCA1* mutation) and KURAMOCHI (*BRCA2* mutation). Among them, Bi2536, a selective PLK1 inhibitor [33], exhibited the most pronounced growth-inhibitory activity, consistently outperforming other compounds across all cell lines tested (Fig. 1D).

Collectively, these findings establish Bi2536 as a broad-spectrum inhibitor of HGSOC proliferation in both PDOs and conventional cell lines, independent of *BRCA* mutational status.

### In vitro antitumor activity of Bi2536 against HGSOC cells

**Fig. 2:**
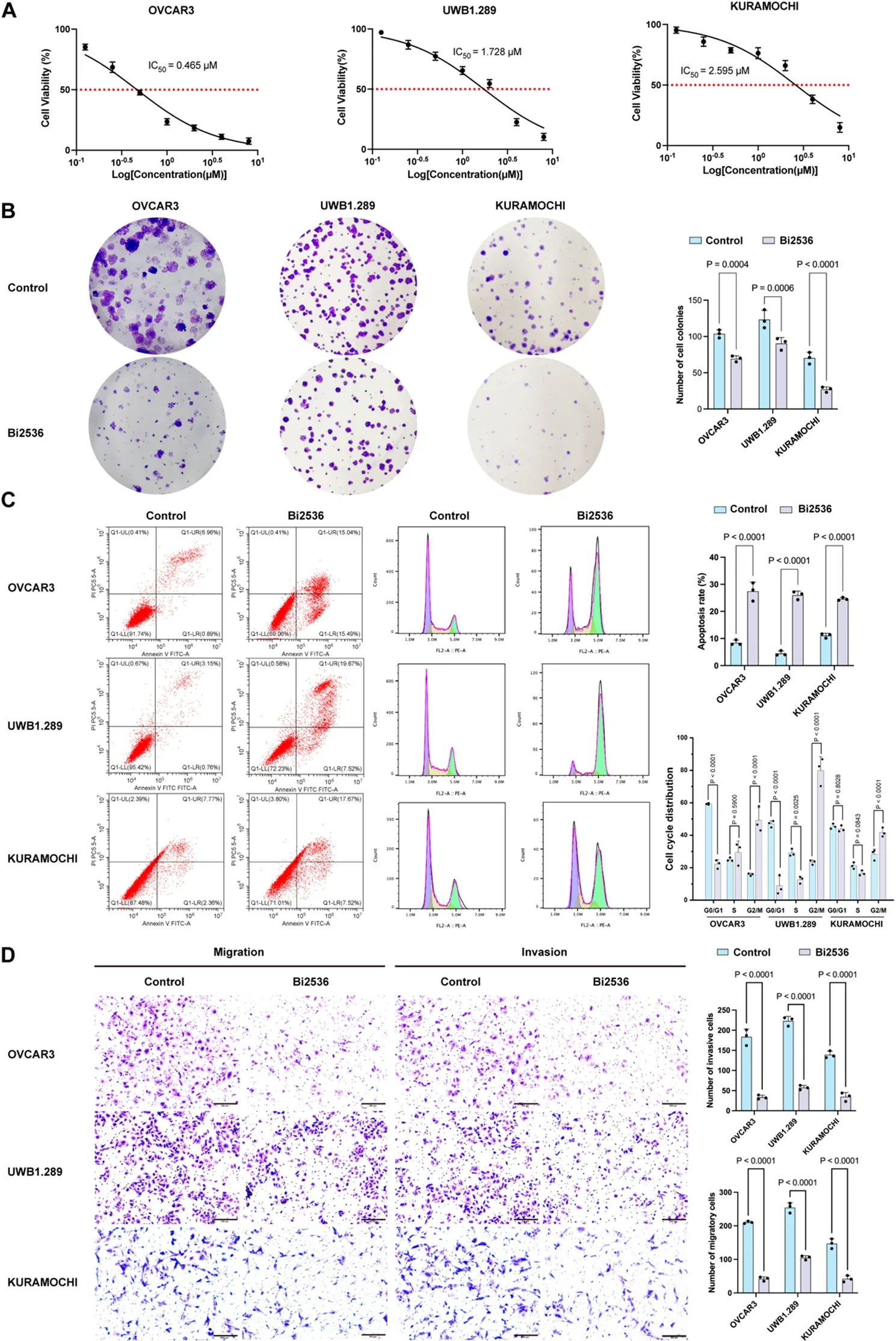
In vitro antitumor activity of Bi2536 against HGSOC cells. (**A**) Dose-response curves of Bi2536 in OVCAR3, UWB1.289, and KURAMOCHI cells after 48 h treatment (CCK-8 assay). (**B**) Inhibition of colony formation by Bi2536 in HGSOC cells. (**C**) Apoptosis and cell-cycle distribution analyzed by flow cytometry. (**D**) Transwell migration and Matrigel-coated invasion assays after 48 h pretreatment with Bi2536 at IC_50_ concentrations; representative images and quantifications are shown (scale bars: 200 μm). Data presentation and statistics: Data are expressed as mean ± SD. Comparisons between two groups were performed using unpaired two-tailed Student’s *t*-test, and comparisons across three groups were analyzed using ANOVA. A P-value < 0.05 was considered statistically significant (specific P-values are indicated in the panels)

To quantitatively assess the in vitro potency of Bi2536 against HGSOC cells, we determined its half-maximal inhibitory concentration (IC_50_) across three genetically diverse cell lines: OVCAR3, UWB1.289, and KURAMOCHI. Bi2536 demonstrated potent growth inhibition with IC_50_ values of 0.465 µM, 1.728 µM, and 2.595 µM, respectively (Fig. 2A). A favorable therapeutic window was revealed by the fact that Bi2536 exhibited the lowest toxicity of IOSE-80 cells on the surface of normal human ovaries at these medication doses (Fig. S3).

We then assessed how Bi2536 affected the main malignant characteristics of OVCAR3, UWB1.289, and KURAMOCHI cells, such as invasion, migration, apoptosis, colony formation, and cell cycle. Cells were first treated with Bi2536 for 48 h at concentrations corresponding to their respective IC_50_ values. Compared with the vehicle control, Bi2536 significantly inhibited colony formation of the three HGSOC cell lines (Fig. 2B). Flow cytometry analysis showed that Bi2536 arrested cells at the G2/M phase and induced apoptosis, which is consistent with the established role of PLK1 in mitotic regulation (Fig. 2C) [34]. Furthermore, the three cell lines’ capacity for invasion and migration was markedly inhibited by Bi2536 treatment (Fig. 2D).

Together, these findings show that Bi2536 inhibits colony formation, migration, and invasion in *BRCA* wild-type and *BRCA*-mutant HGSOC cells while also causing G2/M phase arrest and apoptosis, hence exhibiting wide anticancer action.

### In vivo antitumor efficacy and toxicity of Bi2536 in HGSOC

**Fig. 3:**
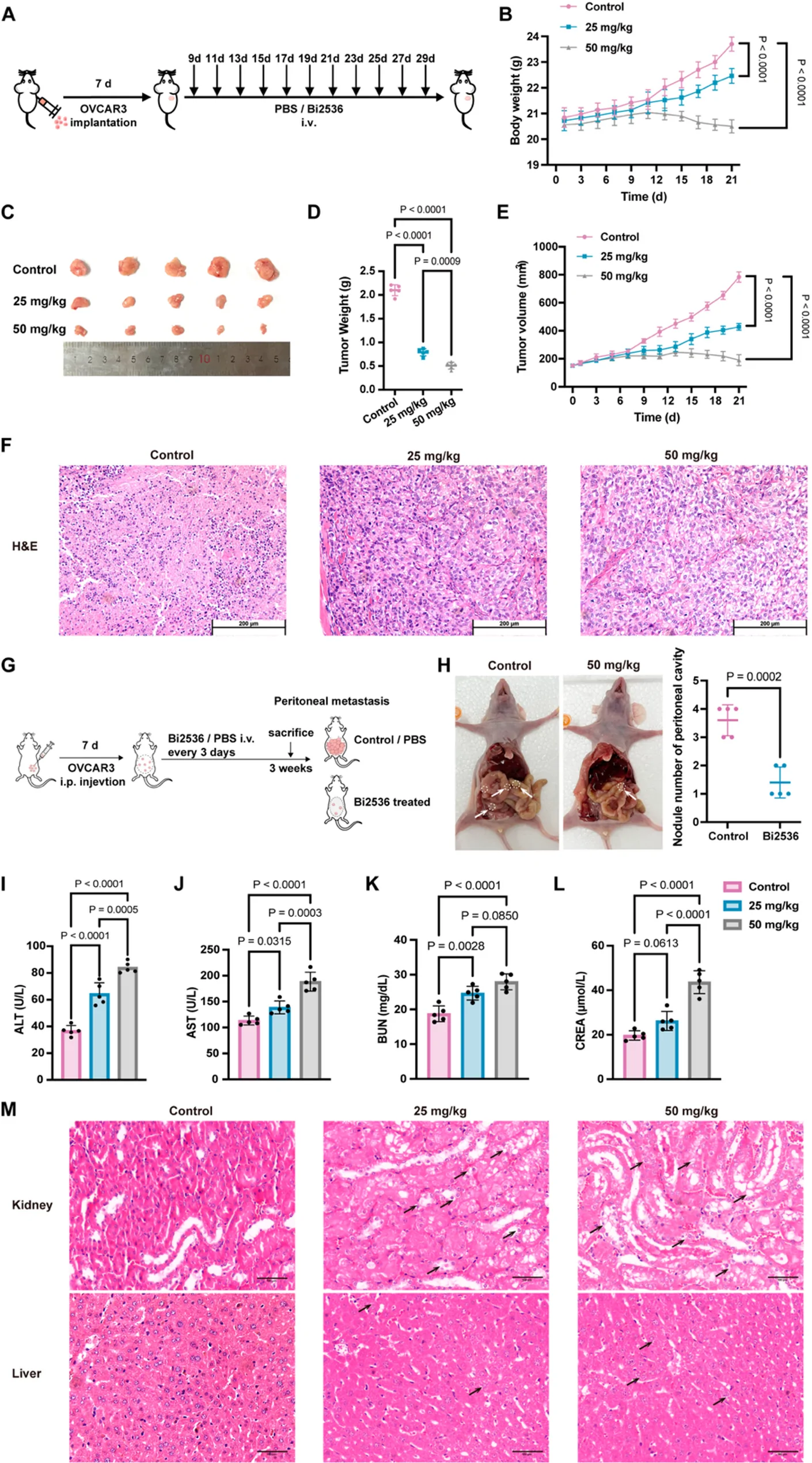
In vivo antitumor efficacy and systemic toxicity of Bi2536 in OVCAR3 xenograft model. (**A**) Schematic of the in vivo experimental design and dosing regimen for vehicle control and Bi2536 (25 or 50 mg/kg). (**B**) Body weight changes during treatment. (**C**) Representative tumor photographs and (**D**) quantified tumor weights at endpoint. (**E**) Tumor growth curves across treatment groups (*n* = 5). (**F**) Representative H&E staining of xenograft tumors (Scale bars: 200 μm). (**G**) Schematic of the in vivo peritoneal metastasis design and dosing regimen for vehicle control and Bi2536 (50 mg/kg). (**H**) Effect of Bi2536 on the number of peritoneal metastases of HGSOC. (**I-L**) Serum biochemical markers of liver (ALT, AST) and kidney (BUN, CREA) function, demonstrating dose-dependent hepatorenal toxicity following free Bi2536 administration. (**M**) Representative H&E staining of liver and kidney tissues after treatment, the arrow indicates degeneration of cells (scale bars: 100 μm)

Subsequently, we evaluated the efficacy of Bi2536 in inhibiting the growth of HGSOC tumors in vivo. To this end, we established HGSOC-CDX models by subcutaneously inoculating OVCAR3, UWB1.289, and KURAMOCHI cells into nude mice. After that, the mice were split into three treatment groups at random and given intravenous infusion of either the carrier control, a low dose of Bi2536 (25 mg/kg), or a high dose of Bi2536 (50 mg/kg) three times a week for four weeks. In OVCAR3-CDX models, Bi2536 treatment induced dose-dependent inhibition of tumor growth, with significantly reduced tumor volume and weight compared to the control group (Fig. 3A-E). Pathological evaluation of resected tumors demonstrated that Bi2536 treatment significantly improved tissue histology by attenuating malignant morphological features. This was corroborated by immunohistochemical analysis, which showed a concurrent decrease in the Ki-67 proliferation index and an increase in activated caspase-3 expression, results that align consistently with our in vitro findings (Fig. 3F, S4). Furthermore, the in vivo antitumor efficacy of Bi2536 was validated in both UWB1.289-CDX and KURAMOCHI-CDX models, with results consistently demonstrating its potent and reproducible activity against HGSOC tumors (Fig. S5-6).

In addition to inhibiting the growth of the primary tumor, we further evaluated the anti-metastasis potential of Bi2536, and reproduced the natural metastasis pathway of HGSOC by using an intraperitoneal diffusion model. After 7 days, mice were randomized into two treatment groups (PBS control and 50 mg/kg Bi2536), followed by tail-vein administration every 3 days for a total of 21 days (Fig. 3G). The frequency and size of intraperitoneal metastatic nodules were significantly lower in the Bi2536-treated group than in the PBS-treated controls (Fig. 3H).

It is worth noting that the temporary weight loss observed in mice treated with a high dose of Bi2536 during tumor growth will lead to an in-depth investigation of serum biochemistry and systemic toxicity of organ histopathology. Serum biochemistry analysis indicated that at 25 mg/kg, levels of alanine aminotransferase (ALT), aspartate aminotransferase (AST), blood urea nitrogen (BUN), and creatinine (CREA) showed slight yet statistically significant elevations. These toxicological effects were markedly exacerbated at the 50 mg/kg dose (Fig. 3I-L). Corroborating the biochemical alterations, histopathological examination revealed dose-dependent organ injuries: hepatocytes displayed mild degenerative changes, while renal tubular epithelial cells exhibited prominent vacuolar degeneration with increasing Bi2536 doses (Fig. 3M).

In a word, these in vivo results show that the dose-dependent antitumor effect of Bi2536 is effective for HGSOC, inhibiting the growth of primary tumor and intraperitoneal metastasis. However, this activity is accompanied by appropriate doses of related systemic toxicity.

### Antitumor mechanisms of Bi2536 in HGSOC cells

Bi2536 inhibitors perform a wide range of antiproliferative activities by inactivating PLK1 and then inhibiting phosphatase in cell cycle division 25 (CDC25). This sustains inhibitory phosphorylation of CDK1 at Tyr15, preventing CDK1/Cyclin B1 complex activation and nuclear translocation. The resulting cytoplasmic sequestration of the inactive complex leads to G2/M arrest and apoptosis, a well-established outcome of PLK1 inhibition [35, 36]. However, the exact molecular mechanism of Bi2536 triggering and inhibiting invasion/migration is still unclear. In order to fill this knowledge vacuum, we thoroughly examined how Bi2536 affected HGSOC cells.

**Fig. 4:**
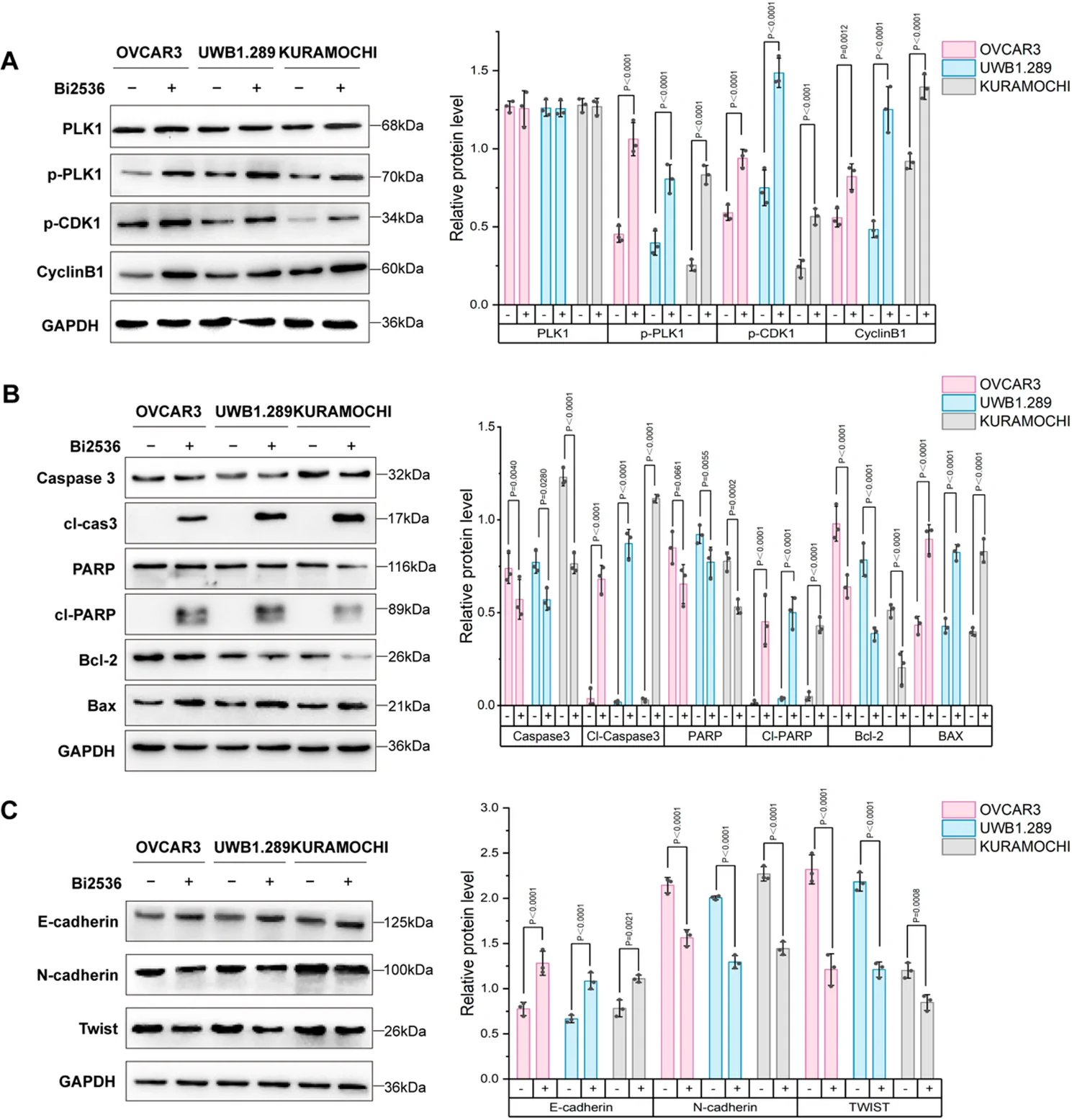
Bi2536 modulates the cell cycle, apoptotic signaling, and EMT program in HGSOC cells. (**A**) Western blot analysis of key G2/M-phase regulators. (**B**) Expression of apoptosis-related proteins in Bi2536-treated HGSOC cells. (**C**) Expression of EMT markers following Bi2536 treatment

To delineate the cell cycle-specific mechanism of Bi2536 in HGSOC, we first analyzed its effect on core cell-cycle regulators. Western blot analysis in OVCAR3, UWB1.289, and KURAMOCHI cells revealed that Bi2536 treatment did not alter total PLK1 protein levels but markedly increased its phosphorylation at the activating T210 residue (p-PLK1[T210]) (Fig. 4A). The accumulation of this inactive phosphorylated PLK1 is consistent with the established mode of action of Bi2536. As an ATP competitive inhibitor, it can stabilize PLK1 in an inactive conformation, allow phosphorylation, and block the automatic dephosphorylation required for activation [13, 37]. Concurrently, we observed canonical markers of mitotic arrest, including sustained inactivation of CDK1 and accumulation of Cyclin B1 (Fig. 4A), confirming that Bi2536 induces a robust G2/M arrest in HGSOC cells, a prerequisite for subsequent apoptotic commitment.

We further dissected the downstream apoptotic cascade initiated by PLK1 inhibition. Bi2536-mediated inactivation of PLK1 locks CDK1 in a persistently phosphorylated, inactive state, precipitating mitotic catastrophe. By dysregulating BCL-2 family proteins, Bi2536 activates the mitochondrial apoptotic pathway. This is accomplished by downregulating the anti-apoptotic protein BCL-2 and upregulating the pro-apoptotic protein BAX. The resulting imbalance disrupts mitochondrial outer membrane integrity, promoting mitochondrial outer membrane permeabilization (MOMP). Consequently, when pro-apoptotic chemicals are released from the mitochondria, caspase 3 is activated, and PARP is cleaved, which are clear indicators of apoptosis [38] (Fig. 4B).

In addition to its pro-apoptotic effects, CDK1 inactivation, which results directly from the disruption of the PLK1-CDK1 axis caused by Bi2536, significantly suppresses the epithelial-mesenchymal transition (EMT), a crucial step in the propagation of HGSOC. Mechanistically, this transcriptional downregulation results from the failure of mitotic exit brought on by CDK1 inactivation. TWIST is a master EMT regulator that typically represses E-cadherin and promotes the expression of mesenchymal markers. Consistent with this model, mitotic arrest induced by Bi2536 (mediated by CDK1 inactivation) reversed the EMT phenomenon, as shown by the up-regulation of E-cadherin (a key invasion inhibitor) and the simultaneous down-regulation of N-cadherin and TWIST (Fig. 4C). This recovery of epithelial characteristics weakens the invasive plasticity of HGSOC cells.

Taken together, our results delineate a coordinated mechanistic cascade: Bi2536 inactivates PLK1 to disrupt the PLK1-CDK1 regulatory axis. The subsequent inactivation of CDK1 then simultaneously triggers mitochondrial apoptosis and blocks the EMT program, thereby elucidating the multifaceted antitumor activity of Bi2536 in HGSOC.

### Circadian oscillation of *PLK1* mRNA expression in HGSOC models

Previous studies have demonstrated that in regenerating mouse liver, the core circadian clock directly regulates WEE1 G2 checkpoint kinase (WEE1) expression, thereby modulating Cyclin B1‑CDK1 kinase activity and establishing a functional link between circadian phase and mitotic control. This, in turn, controls the activity of Cyclin B1-CDK1 kinase [39–41]. Given that PLK1 operates within the same mitotic‑entry axis as WEE1 (WEE1/CDC25‑CDK1‑Cyclin B1), we hypothesized that PLK1 expression might likewise exhibit circadian rhythmicity. To support this hypothesis, a chicken liver circadian time series RNA-seq study combined with weighted gene co-expression network analysis (WGCNA) was performed, which confirmed that *PLK1* was a key gene that might connect circadian rhythm regulation with cell cycle progression. This finding provides a precedent for *PLK1* as a transcriptome‑level output candidate associated with the circadian clock [42].

**Fig. 5:**
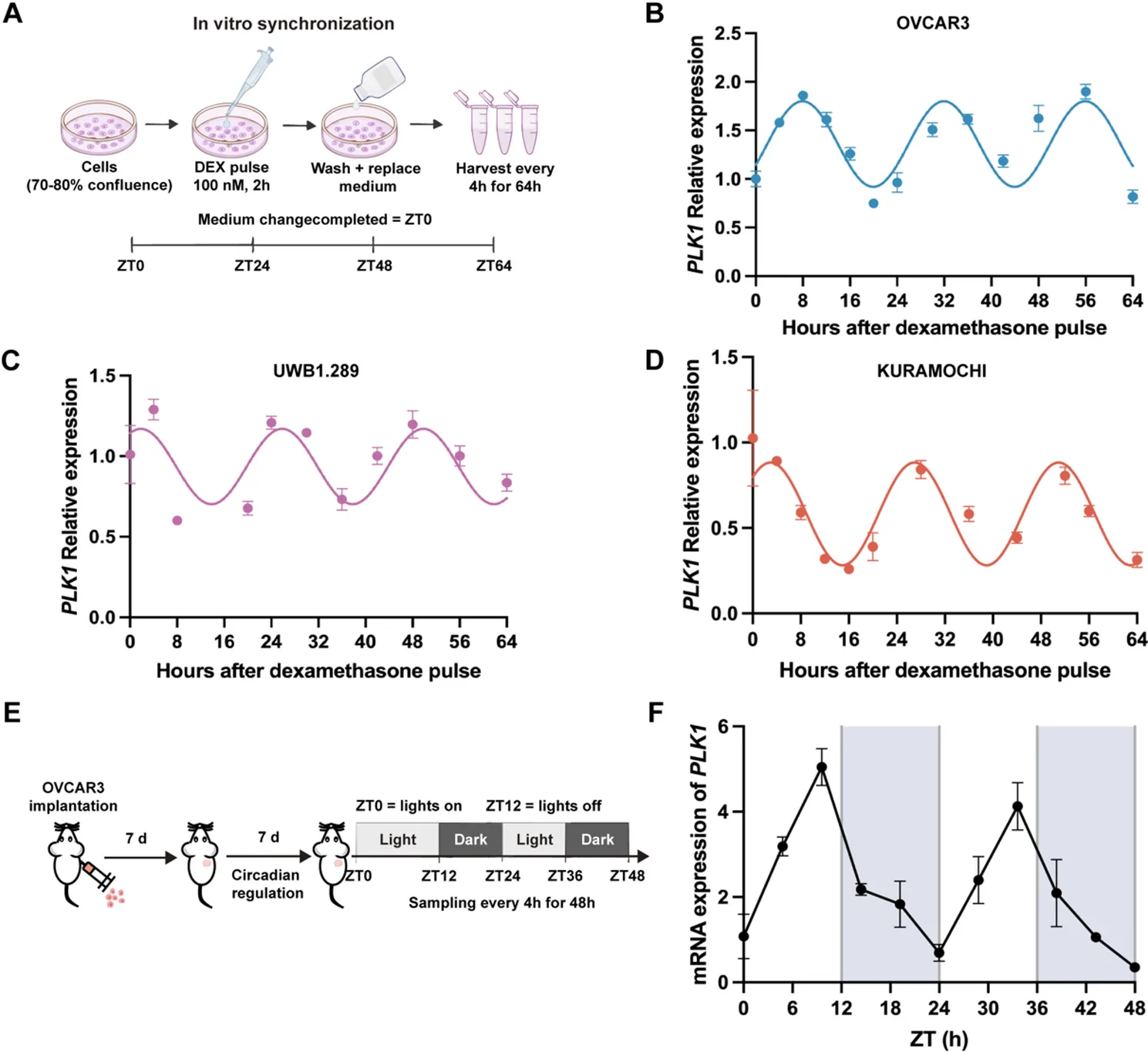
*PLK1* mRNA Expression Displays Circadian Oscillation in HGSOC Models. (**A**) Schematic of intracellular circadian synchronization. (**B‑D**) Temporal expression of *PLK1* mRNA in dexamethasone‑synchronized OVCAR3, UWB1.289, and KURAMOCHI cells. (**E**) Schematic of the in vivo circadian entrainment protocol in xenograft‑bearing mice. (**F**) *PLK1* mRNA levels in OVCAR3 xenograft tumors harvested at different zeitgeber time points under a 12‑h light/12‑h dark cycle

To test this hypothesis, we investigated the temporal dynamics of *PLK1* mRNA expression in HGSOC cells. After synchronizing cell-autonomous circadian rhythms, *PLK1* mRNA levels exhibited reproducible peak-to-trough oscillations over time in OVCAR3, UWB1.289, and KURAMOCHI cells (Fig. 5A-D), confirming that *PLK1* transcription undergoes significant time-dependent variation. We also used OVCAR3 xenograft-bearing mice grown on a typical 12 h light/12 h dark cycle to assess *PLK1* expression in vivo across various zeitgeber periods. Our results showed that *PLK1* mRNA abundance varied significantly with ZT (Fig. 5E, F), demonstrating a clear diurnal oscillation pattern in *PLK1* expression in the tumor tissue.

Collectively, these findings establish a functional link between the core cell-cycle machinery and the molecular circadian oscillator, thereby providing a novel rationale for optimizing PLK1-targeted therapies via chronomodulated drug administration.

### Construction and characterization of biomimetic membrane nanodelivery system

#### Preparation and characterization of HM@TMMSN

**Fig. 6:**
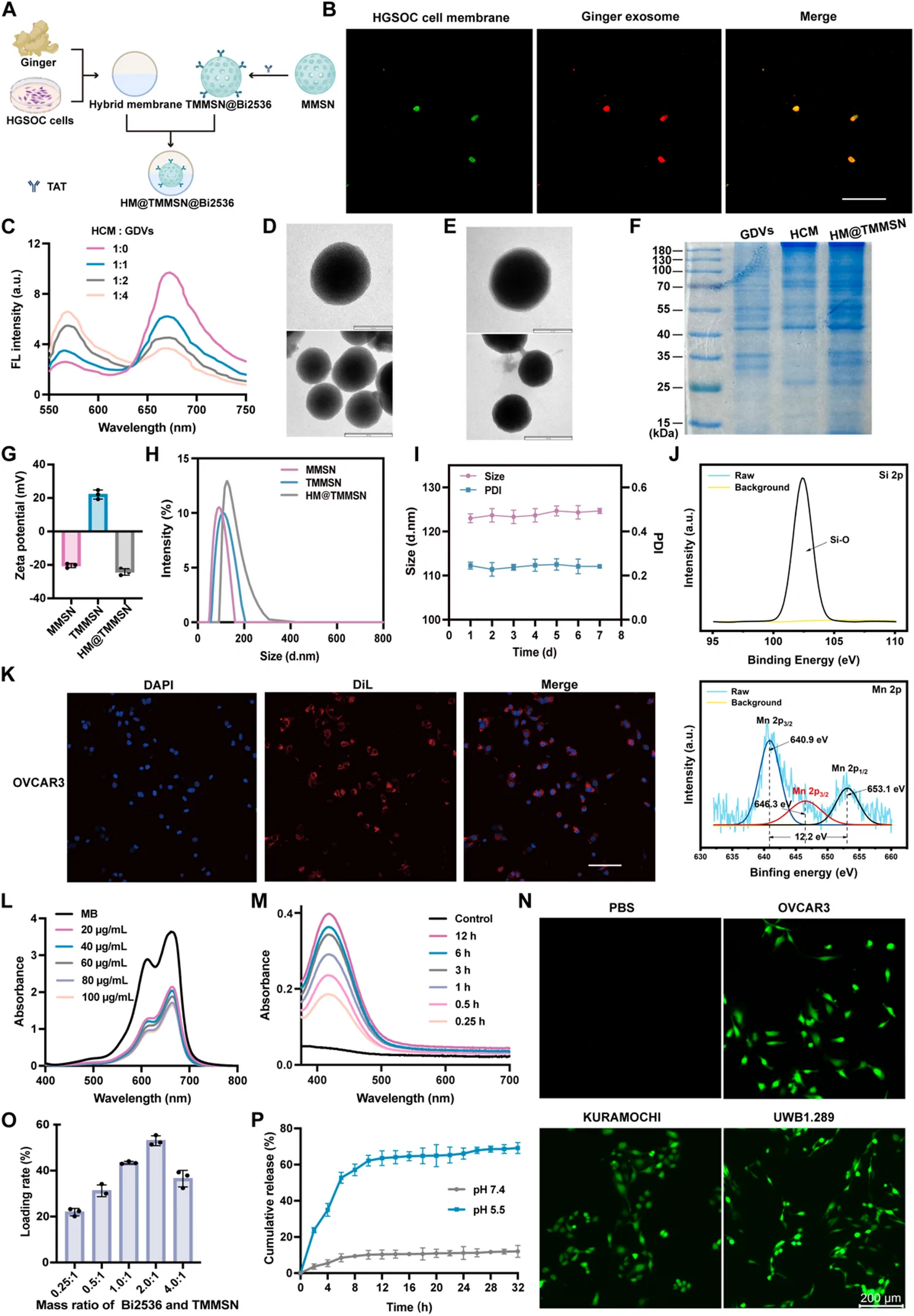
Preparation and characterization of Biomimetic membrane Nanodelivery System. (**A**) Schematic illustration of the formation of HM@TMMSN@Bi2536. Figure created with BioRender.com. (**B**) Confocal images of DiD-labeled GDVs and DiO-labeled HCM after fusion treatments (Scale bars: 100 μm). (**C**) The changes in fluorescence emission of Dil and DiD after cell membrane fusion. (**D, E**) Representative TEM image of TMMSN and HM@TMMSN (Scale bars: 200 μm). (**F**) 10% SDS-PAGE photographs of Marker, GDVs, HCM, and HM@TMMSN. (G, H) Zeta potential and Size distribution of MMSN, TMMSN, HM@TMMSN. (**I**) Stability test of HM@TMMSN. (J) Si 2p XPS spectrum and Mn 2p XPS spectrum of TMMSN nanoparticles. (**K**) CLSM images of OVCAR3 cells cultured with HM@TMMSN NPs for 2 h (Scale bars: 100 μm). (**L**) Absorbance of the MB solution at 665 nm mixed with different concentrations of TMMSN solution. (**M**) UV–vis absorption spectra of OPD mixed with TMMSN solution after treatment in a weakly acidic solution (pH = 5.5) for different times. (**N**) The ability of HM@TMMSN to generate ROS in OVCAR3, KURAMOCHI, and UWB1.289 cells (Scale bars: 200 μm). (**O**) Determination of the loading efficiency of Bi2536. (**P**) Cumulative Bi2536 release profiles

To mitigate Bi2536-induced systemic toxicity and enhance its targeting to HGSOC cells, we synthesized manganese-doped mesoporous silica nanoparticles, modified them with a nuclear-targeting peptide to obtain TMMSN, and further functionalized Bi2536-loaded TMMSN with a biomimetic fusion membrane to form the final nanosystem HM@TMMSN (Fig. 6A). TEM confirmed successful isolation of ginger exosomes with typical morphology (Fig. S7A). Nanoparticle tracking analysis (NTA) results demonstrated that the GDVs exhibited a homogenous size distribution with an average diameter of approximately 143.7 nm and a concentration of 5.5 × 10¹⁰ particles/mL (Fig. S7B). Given that ginger-derived exosomes have been reported to exert antitumor effects in melanoma by Wang et al. [26], we further investigated their antiproliferative activity against HGSOC cells, which showed significant efficacy (Fig. S7C), supporting their potential as a functional component of the fusion membrane. Fusion of HCM and GDVs was initially proved by the use of DiO/DiD labeled fluorescent colocalization (Fig. 6B), and further validated via a FRET assay [43], with increasing HCM: GDVs mass ratios leading to donor (Dil) fluorescence recovery and acceptor (DiD) decrease, confirming successful ultrasonication-induced membrane fusion (Fig. 6C).

The resulting HM was sonicated for 10 min in an ice-water bath to coat the surface of TMMSN in order to prepare HM@TMMSN. Several techniques were used to confirm that HM had been successfully coated. As demonstrated with the TEM images (Fig. 6D, E), TMMSN had a uniform spherical shape, while the cell membranes coated nanocomposites HM@TMMSN, had an obvious core–shell structure, indirectly verifying effective modification by the biomimetic fusion membrane. To verify that HM@TMMSN nanoparticles retain surface-associated antigens from both GDVs and HCM, the protein composition on the surface of HM@TMMSN was analyzed by 10% sodium dodecyl sulfate-polyacrylamide gel electrophoresis (SDS-PAGE). As shown in Fig. 6F, SDS-PAGE results revealed that HM@TMMSN exhibited the corresponding protein bands of both GDVs and HCM, confirming that the relevant membrane proteins of GDVs and HCM were well integrated onto the surface of HM@TMMSN. In contrast, no protein signals were detected in the SDS-PAGE analysis of bare TMMSN (Fig. S8), further validating the successful coating of the HM onto TMMSNs.

Furthermore, dynamic light scattering (DLS) was used to determine the zeta potentials and particle sizes of different nanocomposites. Different from MMSN (-20.3 mV), the zeta potentials of TMMSN and HM@TMMSN were 22.07 mV and − 24.2 mV, respectively (Fig. 6G). The hydrodynamic diameters of TMMSN (107.07 nm) and HM@TMMSN (125.89 nm) were larger than that of MMSN (98.74 nm) (Fig. 6H). In brief, all these results confirm stepwise modification with the nuclear-targeting peptide and fusion membrane. We also examined the stability of various nanocomposites. HM@TMMSN was incubated in PBS containing 10% FBS for 7 days, and the hydrodynamic diameter was recorded by DLS every day. As shown in Fig. 6I, the size and polydispersity index (PDI) of these nanocomposites had negligible changes, proving their good stability, ensuring bloodstream circulation without obvious aggregation, and laying a foundation for in vivo targeted delivery.

Finally, to confirm the elemental composition and chemical state of HM@TMMSNs, XPS analysis was conducted (Fig. 6J). The Si 2p spectrum showed a characteristic peak at 103.4 eV (corresponding to SiO_2_), confirming the silica framework [44]. The Mn 2p spectrum displayed two distinct peaks (Mn 2p_3/2_ at 646.3 eV, Mn 2p_1/2_ at 653.1 eV) with a spin-orbit splitting energy of 12.2 eV, typical of Mn_3_O_4_, confirming the chemical form of doped Mn [45]. Collectively, XPS results validate successful HM@TMMSN synthesis with the designed elemental composition, supporting nanosystem structural integrity.

#### In vitro HGSOC cells targeting

Theoretically, HM@TMMSN could obtain the homologous targeting capability from HGSOC cells, as the functional proteins on HM were well retained on the HM@TMMSN. To verify homologous targeting, three HGSOC cell lines (OVCAR3, UWB1.289, and KURAMOCHI) were incubated with HM@TMMSN NPs for 2 h. CLSM images revealed pronounced red fluorescence derived from HM@TMMSNs accumulated around the nuclei in all three cell types (Fig. 6K and S9). Together, these findings demonstrate the effective HGSOC-targeting ability of HM@TMMSN NPs, resulting in marked perinuclear enrichment and consequently enhanced intranuclear drug delivery.

#### Fenton-like activity of HM@TMMSN NPs for ROS generation

Upon entering the tumor microenvironment, the Mn-O bonds in HM@TMMSN are cleaved under acidic conditions, releasing Mn^2+^ ions. This endows HM@TMMSN with Fenton-like activity, enabling it to catalyze H_2_O_2_ to generate **·**OH for CDT of cancer. As shown in Fig. 6L, the absorbance of MB at 665 nm gradually decreased with increasing nanoparticle concentration, which is attributed to the degradation of MB by ·OH. Similarly, the absorbance of OPD increased progressively with the extension of reaction time (Fig. 6M), confirming the continuous generation of ·OH by HM@TMMSN. Subsequently, the DCFH-DA probe was used to evaluate the ability of HM@TMMSN to generate ROS at the cellular level (Fig. 6N). Cells in the HM@TMMSN group showed high green fluorescence when compared to the PBS control group. This suggests that these nanoparticles can produce ROS in vitro when co-cultured with tumor cells, even in the absence of exogenous H_2_O_2_ supplementation.

#### Drug loading, release profiles, and in vitro cytotoxicity of HM@TMMSN@Bi2536

Given that HM@TMMSNs exhibit favorable physical properties, they hold promise as ideal carriers for antitumor drug delivery. We therefore further loaded Bi2536 into HM@TMMSNs, with the measured LE and EE being 53.02% and 81%, respectively (Fig. 6O and S10). In a tumor microenvironment with simulated acidity (pH 5.5), the cumulative release of HM@TMMSN@Bi2536 was 69.133% (Fig. 6P). HM@TMMSN@Bi2536 exhibited sustained manganese ion release over 48 h at pH 5.5, while only negligible release was detected at pH 7.4. These findings confirm the pH-responsive degradability of the nanocarrier under acidic conditions (Fig. S11). All of these findings demonstrate that nanomaterials have excellent drug loading and pH-responsive release capabilities.

We first used CCK-8 assays to evaluate the in vitro anticancer activity of the HM@TMMSN@Bi2536 formulation and its individual components. As shown in Fig. S12, all tested formulations exhibited concentration-dependent cytotoxicity against OVCAR3 cells. Notably, the HM@TMMSN@Bi2536 formulation exhibited significantly greater cytotoxicity than either the individual components (HM, TMMSN, or Bi2536 alone) or the simple physical mixture (HM+TMMSN+Bi2536). To further validate their synergistic antitumor effects, we conducted live/dead cell staining. As illustrated in Fig. S13, the PBS control group showed high cell viability, whereas the HM@TMMSN@Bi2536 group had the lowest cell survival rate. These findings demonstrate that HM@TMMSN exerts an effective CDT effect and significantly enhances the tumor-killing efficacy of Bi2536.

### In vivo therapeutic efficacy and safety of the biomimetic nanoplatform

**Fig. 7:**
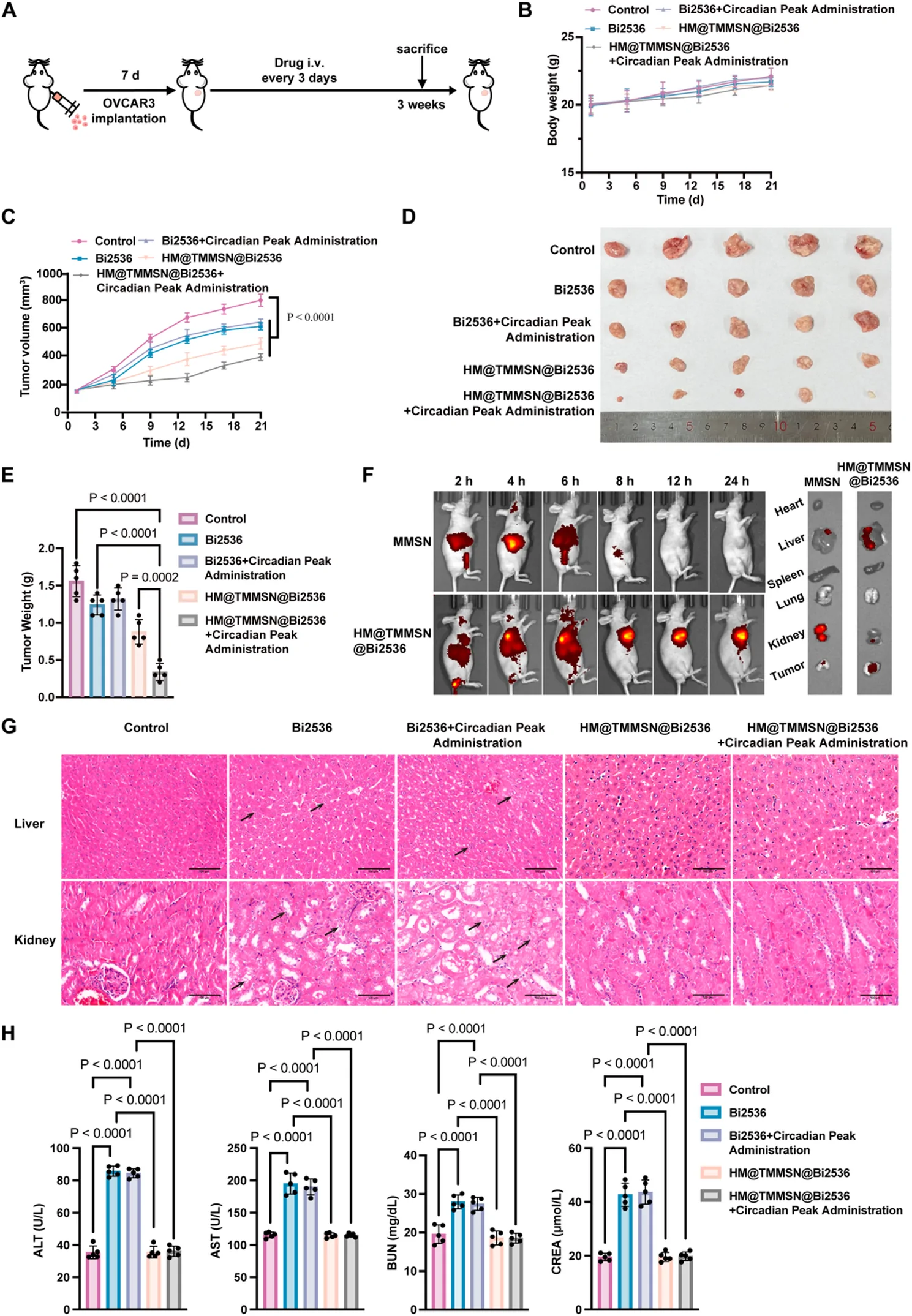
Anti-tumor effect of HM@TMMSN@Bi2536 in a mouse model of HGSOC CDX. (**A**) Schematic illustration of the experimental protocol (*i.v.*, intravenous). Body weight (**B**) and average tumor volume growth curves (**C**) of mice after various treatments (*n* = 5). (**D, E**) Photographs and weight of tumor tissues after excision from different groups of mice at the end of treatments (*n* = 5). (**F**) IVIS images of CDX mice and major organs to evaluate the targeting ability after intravenous injection of HM@TMMSN@Bi2536 and empty MMSN (*n* = 3). (**G**) H&E staining of pathological sections of liver and kidney in each treatment group, the arrow indicates degeneration of cells (Scale bars: 100 μm). (**H**) Serum biochemical analysis of mice following different treatment regimens

We conducted in vivo investigations utilizing the OVCAR3 xenograft model to examine the potential of the bionic nanoplatform to improve therapeutic efficacy and lower toxicity by combining spatiotemporal drug delivery with synergistic CDT. Based on preliminary dose-finding experiments (0, 25, and 50 mg/kg), a Bi2536 dose of 50 mg/kg was selected, as it exhibited a favorable tumor-inhibitory capacity while being limited by toxicity in its free form. As indicated by the flowchart in Fig. 7A, mice were randomly assigned to one of five treatment groups: control, free Bi2536, free Bi2536 administered at the circadian peak (ZT10), HM@TMMSN@Bi2536, and HM@TMMSN@Bi2536 administered at ZT10. The drug was injected intravenously every three days for 21 consecutive days. The results showed that all the treatment groups maintained a relatively stable weight trajectory during the treatment (Fig. 7B). Tumor volume monitoring revealed significant differences in therapeutic efficacy among groups (Fig. 7C). Free Bi2536 exerted moderate tumor growth inhibition, while HM@TMMSN encapsulation significantly enhanced tumor suppression, attributed to improved tumor targeting and reduced off-target toxicity of the nanoplatform. Interestingly, anticancer activity was further enhanced by administration at ZT10, which corresponds to the highest *PLK1* expression found in our earlier circadian rhythm research. This synergistic effect ultimately led to the most significant reduction in tumor burden in the HM@TMMSN@Bi2536 + ZT10 group, confirmed by final tumor weights (Fig. 7E) and representative images of excised tumors (Fig. 7D). Further IHC analysis of PLK1 signaling indicated that total PLK1 expression remained unchanged, whereas phosphorylated PLK1 (p-PLK1) levels were elevated in Bi2536-treated tumors (Fig. S14). This result is consistent with the data in vitro, and it is confirmed that Bi2536 inactivates PLK1 specifically instead of changing the total expression of PLK1. These results confirm that the combination of bionic nanoparticle delivery and circadian rhythm time administration optimizes the therapeutic effect of Bi2536.

We further verify the homologous target ability of the biomimetic nanoparticles delivery system through in vivo fluorescence imaging. As shown in Fig. 7F, HM@TMMSN exhibited enhanced tumour accumulation at 8 h post-injection compared with bare MMSN, verifying its improved tumour-targeting capability. Considering the time-dependent processes of nanoparticle enrichment and subsequent drug release, we performed preemptive administration to match the intrinsic circadian rhythm of *PLK1*. Given that endogenous *PLK1* expression in tumor was found to peak at ZT10, HM@TMMSN@Bi2536 was intravenously delivered at ZT2, 8 h ahead of the *PLK1* rhythmic peak, to allow sufficient time for tumour accumulation and drug release. Subsequent LC-MS/MS quantification of free Bi2536 in tumor tissues collected from ZT4 to ZT16 showed that the drug concentration peaked precisely at ZT10, and the intratumor concentration of Bi2536 was about 263.1ng/mg (Fig. S15), perfectly coinciding with the *PLK1* maximum. This confirms that pre-administration at ZT2 effectively synchronizes the peak drug release with the *PLK1* rhythm, thereby validating the rationale of our timed chronotherapy strategy.

Histological analysis of tumor samples was used to further assess the treatment efficacy of various administration groups. H&E evaluation of the free Bi2536 group showed evident kidney damage and minor liver injury, including hepatocellular vacuolar degeneration, as well as renal tubular injury (Fig. 7G). In sharp contrast, mice treated with HM@TMMSN@Bi2536 exhibited no discernible pathological abnormalities in the liver or kidney (Fig. 7G). Serum biochemical indicators corroborate these findings: the HM@TMMSN@Bi2536 group’s ALT/AST and BUN/Crea levels are comparable to those of the control group, whereas the free Bi2536 group’s levels are noticeably higher (Fig. 7H). Moreover, the biosafety of nanomaterials was verified by a hemolysis assay. According to the results, HM@TMMSN and HM@TMMSN@Bi2536 NPs did not appear to cause any hemolysis, indicating their high biocompatibility and potential for in vivo use (Fig. S16). Overall, these data confirmed that HM@TMMSN packaging Bi2536 successfully separated the therapeutic effect of Bi2536 from its non-target organ toxicity, and PLK1 peak treatment management (ZT10) further optimized the treatment window.

## Discussion

HGSOC remains the most prevalent and lethal subtype of ovarian cancer, with a five-year survival rate of merely 49.1% globally, largely due to intricate drug resistance mechanisms and limited effective therapeutic options [46–48]. There are unmet clinical needs because PARP inhibitors have not helped the majority of patients with *BRCA* wild-type malignancies, despite their considerable clinical success in HGSOC patients with *BRCA1/2* mutation. This therapeutic disparity stems from the reliance of PARP inhibitors on synthetic lethality with defective homologous recombination repair (HRR) mediated by *BRCA* mutations, leaving HRR-proficient *BRCA* wild-type tumors unaffected. In order to fill this gap, we use PDO representing different *BRCA* states (mutant and wild type) as a clinically relevant screening platform, and more accurately summarize the heterogeneity and biological characteristics of primary HGSOC tumors, instead of traditional cell lines.

Our screening and validation results demonstrated that Bi2536, a potent PLK1 inhibitor, exerts robust antitumor effects against HGSOC across all *BRCA* statuses, overcoming the inherent limitation of PARP inhibitors [49–51]. This finding is particularly significant given the paucity of research on Bi2536 in HGSOC, with prior studies focusing primarily on other tumor types such as small cell lung cancer. Our findings, however, demonstrate that Bi2536’s curative effect in HGSOC is unaffected by *BRCA* status, underscoring the drug’s promise as a universal treatment agent for this refractory condition. This independence from *BRCA* status suggests that Bi2536 targets a core biological process essential for HGSOC cell survival, irrespective of HRR competency, thereby bypassing the resistance mechanisms that limit PARP inhibitor utility.

To fill the current information gap about PLK1 inhibition in HGSOC, we carried out comprehensive studies into Bi2536’s mode of action in order to clarify the underlying molecular mechanisms. Our results delineate a clear and sequential signaling cascade: Bi2536 first induces direct inactivation of PLK1, and this PLK1 inactivation subsequently leads to downstream inactivation of CDK1 suppression directly triggers G2/M phase arrest [11, 12]—an effect that prevents HGSOC cells from progressing through mitosis and initiating cell division, it is accompanied by the induction of apoptotic cell death and the impairment of cell invasion and migration, both of which are key hallmarks of HGSOC progression and metastasis. Notably, this entire molecular mechanism is consistent across both *BRCA* mutant and wild-type HGSOC models, which directly explains the broad efficacy of Bi2536 independent of *BRCA* status. Bi2536 plays an anti-tumor role by inducing cell cycle arrest, apoptosis and EMT arrest, which is especially relevant in HGSOC where metastasis and drug resistance are the main drivers of mortality.

During the course of our studies, we made a novel observation: *PLK1* expression exhibits inherent circadian rhythmicity in HGSOC cells [52]. Circadian rhythms are governed by a core transcriptional-translational feedback loop involving clock genes such as *Brain and Muscle ARNT-Like 1 (BMAL1)*, *Circadian Locomotor Output Cycles Kaput (CLOCK)*, *Period (PER)*, *and Cryptochrome (CRY)*, which regulate the expression of numerous downstream genes involved in cell cycle progression and tumorigenesis [53–57]. However, the molecular basis of *PLK1*’s circadian rhythmicity in HGSOC remains poorly defined, representing a critical limitation of our current work. Future research should be aimed at filling this gap by checking whether the basic clock molecules directly regulate the expression of *PLK1*. For example, it can be determined whether the circadian rhythm of *PLK1* is cancelled for key clock genes (e.g., *BMAL1* or *CLOCK*), thus providing an overview of the circadian rhythm biology and the regulatory network of *PLK1* function in HGSOC. Additionally, further research is needed to explore whether this circadian rhythmicity influences Bi2536’s efficacy, as timing of drug administration relative to *PLK1*’s expression peak could potentially enhance therapeutic outcomes.

We suggest a unique therapeutic approach that combines time-dependent dosing, nanomaterial-mediated synergistic therapy, and Bi2536 in order to apply these findings to practical practice. Nanomaterials have emerged as promising drug delivery systems for cancer treatment, particularly for enhancing the efficacy of chemotherapy and CDT. CDT relies on Fenton or Fenton-like reactions to convert endogenous H_2_O_2_ into highly toxic ·OH, inducing irreversible cellular damage [58–61]. Nevertheless, excessive intracellular glutathione (GSH) in tumor cells might neutralize ·OH, decreasing the efficacy of CDT. In order to produce a synergistic therapeutic effect, nanomaterials can be designed to administer CDT agents and Bi2536 (chemotherapy) simultaneously while also scavenging GSH to increase CDT efficacy [62, 63]. Furthermore, integrating this nanomaterial-based combination therapy with time-of-day dosing aligned with PLK1’s circadian peak could maximize Bi2536’s antitumor effects while minimizing off-target toxicity to normal tissues, which also exhibit circadian variations in cell cycle activity [14]. This method solves the limitation of single drug therapy, and uses molecular targeting and circadian biology to optimize the treatment results, which is an important consideration of HGSOC, because the treatment window is usually narrow.

In conclusion, our study identifies Bi2536 as a promising universal therapeutic agent for HGSOC, effective across all *BRCA* statuses, and delineates its mechanism of action via PLK1-CDK1 inactivation-mediated G2/M arrest, apoptosis, and reduced metastatic potential. We also report the novel observation of *PLK1*’s circadian rhythmicity in HGSOC, highlighting a new area for future research. Finally, we propose a innovative combinatorial strategy integrating nanomaterial-mediated chemotherapy-CDT synergy and time-dependent dosing to enhance Bi2536’s clinical efficacy. Although our results offer a solid basis, more preclinical and clinical research is required to confirm the safety and effectiveness of this strategy, especially the function of circadian clock molecules in controlling PLK1 expression and the translational potential of delivery systems based on nanomaterials. Ultimately, this work aims to address the unmet clinical need for effective, broadly applicable therapies for HGSOC, improving outcomes for all patients regardless of *BRCA* status.

## Conclusion

In summary, we delineated that the PLK1 inhibitor Bi2536 exerts anti-tumor effects in HGSOC by inactivating PLK1, thereby inducing G2/M phase arrest, mitochondrial apoptosis, and suppressing EMT. It is worth noting that *PLK1* exhibits circadian expression dynamics, and integrating Bi2536 with a biomimetic nano-delivery system (constructed by fusing ginger exosomes with HGSOC cell membranes) and circadian-timed administration, which reduces the normal toxicity of tissues, improves the efficiency through the synergistic effect of chemotherapy-CDT, and is expected to become a new treatment paradigm of HGSOC.

## Supplementary Information


Supplementary Material 1


## Data Availability

No datasets were generated or analysed during the current study.

## References

[1] Bowtell DD, Böhm S, Ahmed AA, Aspuria PJ, Bast RC Jr, Beral V, Berek JS, Birrer MJ, Blagden S, Bookman MA, et al. Rethinking ovarian cancer II: reducing mortality from high-grade serous ovarian cancer. Nat Rev Cancer. 2015;15:668–79. 26493647 10.1038/nrc4019 PMC4892184

[2] Prat J. Ovarian carcinomas: five distinct diseases with different origins, genetic alterations, and clinicopathological features. Virchows Arch. 2012;460:237–49. 22322322 10.1007/s00428-012-1203-5

[3] Anonymous. Epithelial ovarian cancer: evolution of management in the era of precision medicine. CA Cancer J Clin. 2019;69:280–304. 31099893 10.3322/caac.21559

[4] Cancer Genome Atlas Research Network. Integrated genomic analyses of ovarian carcinoma. Nature. 2011;474:609–15. 21720365 10.1038/nature10166 PMC3163504

[5] Ciriello G, Miller ML, Aksoy BA, Senbabaoglu Y, Schultz N, Sander C. Emerging landscape of oncogenic signatures across human cancers. Nat Genet. 2013;45:1127–33. 24071851 10.1038/ng.2762 PMC4320046

[6] Jayson GC, Kohn EC, Kitchener HC, Ledermann JA. Ovarian cancer. Lancet. 2014;384:1376–88. 24767708 10.1016/S0140-6736(13)62146-7

[7] Semertzidou A, Brosens JJ, McNeish I, Kyrgiou M. Organoid models in gynaecological oncology research. Cancer Treat Rev. 2020;90:102103. 32932156 10.1016/j.ctrv.2020.102103

[8] Siegel RL, Miller KD, Fuchs HE, Jemal A. Cancer statistics, 2021. CA Cancer J Clin. 2021;71:7–33. 33433946 10.3322/caac.21654

[9] Huang T, Huang W, Bian Q. Organoids as Predictive Platforms: Advancing Disease Modeling, Therapeutic Innovation, and Drug Delivery Systems. J Control Release. 2025;387:114222. 40939865 10.1016/j.jconrel.2025.114222

[10] Sachs N, de Ligt J, Kopper O, Gogola E, Bounova G, Weeber F, Balgobind AV, Wind K, Gracanin A, Begthel H, et al. A Living Biobank of Breast Cancer Organoids Captures Disease Heterogeneity. Cell. 2018;172:373–e38610. 29224780 10.1016/j.cell.2017.11.010

[11] Shen X, Wang J, Chen S, Zhang H, Zhou C, Bae-Jump VL. Clinical relevance of PLK1 in epithelial ovarian cancer. Front Pharmacol. 2025;16:1702273. 41458961 10.3389/fphar.2025.1702273 PMC12741083

[12] Conti D, Verza AE, Pesenti ME, Cmentowski V, Vetter IR, Pan D, Musacchio A. Role of protein kinase PLK1 in the epigenetic maintenance of centromeres. Science. 2024;385:1091–7. 39236163 10.1126/science.ado5178

[13] Choi M, Kim W, Cheon MG, Lee CW, Kim JE. Polo-like kinase 1 inhibitor Bi2536 causes mitotic catastrophe following activation of the spindle assembly checkpoint in non-small cell lung cancer cells. Cancer Lett. 2015;357:591–601. 25524551 10.1016/j.canlet.2014.12.023

[14] Sebastian M, Reck M, Waller CF, Kortsik C, Frickhofen N, Schuler M, Fritsch H, Gaschler-Markefski B, Hanft G, Munzert G, von Pawel J. The efficacy and safety of Bi2536, a novel Plk-1 inhibitor, in patients with stage IIIB/IV non-small cell lung cancer who had relapsed after, or failed, chemotherapy: results from an open-label, randomized phase II clinical trial. J Thorac Oncol. 2010;5(7):1060–7. 20526206 10.1097/JTO.0b013e3181d95dd4

[15] Pandha HS, Protheroe A, Wylie J, Parker C, Chambers J, Bell S, Munzert G. An open label phase II trial of Bi2536, a novel Plk1 inhibitor, in patients with metastatic hormone refractory prostate cancer (HRPC). J Clin Oncol. 2008;26(15suppl):14547.

[16] Von Pawel J, Reck M, Digel W, Kortsik C, Thomas M, Frickhofen N, Schuler M, Gaschler-Markefski B, Hanft G, Sebastian M. Randomized phase II trial of two dosing schedules of Bi2536, a novel Plk-1 inhibitor, in patients with relapsed advanced or metastatic non-small-cell lung cancer (NSCLC). J Clin Oncol. 2008;26(15suppl):8030.

[17] Mross K, Dittrich C, Aulitzky WE, Strumberg D, Schutte J, Schmid RM, Hollerbach S, Merger M, Munzert G, Fleischer F, Scheulen ME. A randomised phase II trial of the polo-like kinase inhibitor Bi2536 in chemo-naïve patients with unresectable exocrine adenocarcinoma of the pancreas—a study within the central european society anticancer drug research (CESAR) collaborative network. Br J Cancer. 2012;107(2):280–6. 22699824 10.1038/bjc.2012.257 PMC3394983

[18] Sulli G, Lam MTY, Panda S. Interplay between circadian clock and cancer: new frontiers for cancer treatment. Trends Cancer. 2019;5(8):475–94. 31421905 10.1016/j.trecan.2019.07.002 PMC7120250

[19] Sulli G, Manoogian ENC, Taub PR, Panda S. Training the circadian clock, clocking the drugs, and drugging the clock to prevent, manage, and treat chronic diseases. Trends Pharmacol Sci. 2018;39(9):812–27. 30060890 10.1016/j.tips.2018.07.003 PMC7249726

[20] Hanahan D, Weinberg RA. Hallmarks of cancer: the next generation. Cell. 2011;144(5):646–74. 21376230 10.1016/j.cell.2011.02.013

[21] Karaboué A, Innominato PF, Wreglesworth NI, Duchemann B, Adam R, Lévi FA. Why does circadian timing of administration matter for immune checkpoint inhibitors’ efficacy? Br J Cancer. 2024;131(5):783–96. 38834742 10.1038/s41416-024-02704-9 PMC11369086

[22] Qian DC, Kleber T, Brammer B, Xu KM, Switchenko JM, Janopaul–Naylor JR, Zhong J, Yushak ML, Harvey RD, Paulos CM, Lawson DH, Khan MK, Kudchadkar RR, Buchwald ZS. Effect of immunotherapy time–of–day infusion on overall survival among patients with advanced melanoma in the USA (MEMOIR): a propensity score–matched analysis of a single–centre, longitudinal study. Lancet Oncol. 2021;22(12):1777–86. 34780711 10.1016/S1470-2045(21)00546-5

[23] Tembo KM, Wang X, Bolideei M, et al. Exploring the bioactivity of MicroRNAs Originated from Plant-derived Exosome-like Nanoparticles (PELNs): current perspectives. J Nanobiotechnol. 2025;23:563. 10.1186/s12951-025-03602-9 PMC12344895 40796868

[24] Han R, Zhou D, Ji N, et al. Folic acid-modified ginger-derived extracellular vesicles for targeted treatment of rheumatoid arthritis by remodeling immune microenvironment via the PI3K-AKT pathway. J Nanobiotechnol. 2025;23:41. 10.1186/s12951-025-03096-5 PMC11756199 39849554

[25] Li S, Zhang R, Wang A, Li Y, Zhang M, Kim J, Zhu Y, Wang Q, Zhang Y, Wei Y, Wang J. Panax notoginseng–derived exosome–like nanoparticles attenuate ischemia reperfusion injury via altering microglia polarization. J Nanobiotechnol. 2023;21:416. 10.1186/s12951-023-02161-1 PMC10636993 37946257

[26] Wang F, Li LY, Deng JY, Ai JC, Mo S, Ding DD, Xiao YX, Hu S, Zhu D, Li QS, Zeng Y, Chen ZT, Cheng K, Li ZH. Lipidomic analysis of plant–derived extracellular vesicles for guidance of potential anti–cancer therapy. Bioact Mater. 2024;46:82–96. 39737211 10.1016/j.bioactmat.2024.12.001 PMC11683192

[27] Sundaram K, Mu J, Kumar A, Behera J, Lei C, Sriwastva MK, Xu F, Dryden GW, Zhang L, Chen SY, Yan J, Zhang X, Park JW, Merchant ML, Tyagi N, Teng Y, Zhang HG. Garlic exosome–like nanoparticles reverse high–fat diet–induced obesity via the gut/brain axis. Theranostics. 2022;12:1220–46. 35154484 10.7150/thno.65427 PMC8771565

[28] Lei C, Mu J, Teng Y, He L, Xu F, Zhang X, Sundaram K, Kumar A, Sriwastva MK, Lawrenz MB, Zhang L, Yan J, Feng W, McClain CJ, Zhang X, Zhang HG. Lemon exosome–like nanoparticles–manipulated probiotics protect mice from C. diff infection. iScience. 2020;23:101571. 33083738 10.1016/j.isci.2020.101571 PMC7530291

[29] Lin LS, Song J, Song L, Ke K, Liu Y, Zhou Z, Shen Z, Li J, Yang Z, Tang W, Niu G, Yang HH, Chen X. Simultaneous Fenton–like ion delivery and glutathione depletion by MnO₂–based nanoagent to enhance chemodynamic therapy. Angew Chem Int Ed Engl. 2018;57:4902–6. 29488312 10.1002/anie.201712027

[30] Liu B, Chen X, Zhu Y, Chen H, Tan J, Yang Z, Li J, Zheng P, Feng L, Wang Q, Gai S, Zhong L, Yang P, Cheng Z, Lin J. One–step symbiosis of bimetallic peroxides nanoparticles to induce ferroptosis/cuproptosis and activate cGAS–STING pathway for enhanced tumor immunotherapy. Adv Mater. 2025;37:e2500337. 40181655 10.1002/adma.202500337

[31] Boretto M, Maenhoudt N, Luo X, Hennes A, Boeckx B, Bui B, Heremans R, Perneel L, Kobayashi H, Van Zundert I, et al. Patient-derived organoids from endometrial disease capture clinical heterogeneity and are amenable to drug screening. Nat Cell Biol. 2019;21:1041–51. 31371824 10.1038/s41556-019-0360-z

[32] Cathro HP, Stoler MH. Expression of cytokeratins 7 and 20 in ovarian neoplasia. Am J Clin Pathol. 2002;117:944–51. 12047147 10.1309/2T1Y-7BB7-DAPE-PQ6L

[33] von Glasenapp V, Almeida AC, Chang D, Gasic I, Winssinger N, Gotta M. Spatio-temporal control of mitosis using light via a PLK1 inhibitor caged for activity and cellular permeability. Nat Commun. 2025;16:1599. 39971898 10.1038/s41467-025-56746-5 PMC11840123

[34] Zhang J, Li Z, Xie MY, Wang N, Liu H, Li F, Pang LJ, Qi Y. Overexpression of oncogenic polo-like kinase 1 disrupts the invasiveness, cell cycle, and apoptosis in synovial sarcoma. J Transl Med. 2025;23:691. 40542346 10.1186/s12967-025-06740-8 PMC12181926

[35] Steegmaier M, Hoffmann M, Baum A, Lénárt P, Petronczki M, Krssák M, Gürtler U, Garin-Chesa P, Lieb S, Quant J, Grauert M, Adolf GR, Kraut N, Peters JM, Rettig WJ. Bi2536, a potent and selective inhibitor of polo-like kinase 1, inhibits tumor growth in vivo. Curr Biol. 2007;17:316–22. 17291758 10.1016/j.cub.2006.12.037

[36] Li Z, Yang C, Li X, Du X, Tao Y, Ren J, Fang F, Xie Y, Li M, Qian G, Xu L, Cao X, Wu Y, Lv H, Hu S, Lu J, Pan J. The dual role of Bi2536, a small-molecule inhibitor that targets PLK1, in induction of apoptosis and attenuation of autophagy in neuroblastoma cells. J Cancer. 2020;11:3274–87. 32231733 10.7150/jca.33110 PMC7097946

[37] Li HY, Luo F, Li XY, Fu XF, He JF, Tian YZ, Zhu JJ, Chu XY, Zhao HL. Inhibition of polo-like kinase 1 by Bi2536 reverses the multidrug resistance of human hepatoma cells in vitro and in vivo. Anticancer Agents Med Chem. 2019;19:740–9. 30836927 10.2174/1871520619666190301145637

[38] Wu M, Wang Y, Yang D, Gong Y, Rao F, Liu R, Danna Y, Li J, Fan J, Chen J, Zhang W, Zhan Q. A PLK1 kinase inhibitor enhances the chemosensitivity of cisplatin by inducing pyroptosis in oesophageal squamous cell carcinoma. EBioMedicine. 2019;41:244–55. 30876762 10.1016/j.ebiom.2019.02.012 PMC6442225

[39] Mello RM, Gomez Ceballos D, Sandate CR, Wang S, Jouffe C, Agudelo D, Uhlenhaut NH, Thomä NH, Simon MC, Lamia KA. BMAL1 and ARNT enable circadian HIF2α responses in clear cell renal cell carcinoma. Nat Commun. 2025;16:5834. 40595592 10.1038/s41467-025-60904-0 PMC12215836

[40] Hunt T, Sassone-Corsi P. Riding tandem: Circadian clocks and the cell cycle. Cell. 2007;129:461–4. 17482541 10.1016/j.cell.2007.04.015

[41] Matsuo T, Yamaguchi S, Mitsui S, Emi A, Shimoda F, Okamura H. Control mechanism of the circadian clock for timing of cell division in vivo. Science. 2003;302:255–9. 12934012 10.1126/science.1086271

[42] Wang P, Li F, Sun Y, Li Y, Xie X, Du X, Liu L, Wu Y, Song D, Xiong H, Chen J, Li X. Novel insights into the circadian modulation of lipid metabolism in chicken livers revealed by RNA sequencing and weighted gene co-expression network analysis. Poult Sci. 2024;103:104321. 39361997 10.1016/j.psj.2024.104321 PMC11474196

[43] Zhang X, Hu Y, Yang X, Tang Y, Han S, Kang A, Deng H, Chi Y, Zhu D, Lu Y. Förster resonance energy transfer (FRET)-based biosensors for biological applications. Biosens Bioelectron. 2019;138:111314. 31096114 10.1016/j.bios.2019.05.019

[44] Tan D, Ma Z, Xu B, Dai Y, Ma G, He M, Jin Z, Qiu J. Surface passivated silicon nanocrystals with stable luminescence synthesized by femtosecond laser ablation in solution. Phys Chem Chem Phys. 2011;13:20255–61. 21993573 10.1039/c1cp21366k

[45] Masa J, Xia W, Sinev I, Zhao A, Sun Z, Grützke S, Weide P, Muhler M, Schuhmann W. Mn(x)O(y)/NC and Co(x)O(y)/NC nanoparticles embedded in a nitrogen-doped carbon matrix for high-performance bifunctional oxygen electrodes. Angew Chem Int Ed Engl. 2014;53:8508–12. 24975388 10.1002/anie.201402710

[46] Bowtell DD, Böhm S, Ahmed AA, Aspuria PJ, Bast RC Jr, Beral V, Berek JS, Birrer MJ, Blagden S, Bookman MA, Brenton JD, Chiappinelli KB, Martins FC, Coukos G, Drapkin R, Edmondson R, Fotopoulou C, Gabra H, Galon J, Gourley C, Heong V, Huntsman DG, Iwanicki M, Karlan BY, Kaye A, Lengyel E, Levine DA, Lu KH, McNeish IA, Menon U, Narod SA, Nelson BH, Nephew KP, Pharoah P, Powell DJ Jr, Ramos P, Romero IL, Scott CL, Sood AK, Stronach EA, Balkwill FR. Rethinking ovarian cancer II: reducing mortality from high-grade serous ovarian cancer. Nat Rev Cancer. 2015;15:668–79. 26493647 10.1038/nrc4019 PMC4892184

[47] Ju HY, Youn SY, Kang J, Whang MY, Choi YJ, Han MR. Integrated analysis of spatial transcriptomics and CT phenotypes for unveiling the novel molecular characteristics of recurrent and non-recurrent high-grade serous ovarian cancer. Biomark Res. 2024;12:80. 39135097 10.1186/s40364-024-00632-7 PMC11318304

[48] Carey KM, Young CD, Clark AJ, Dammer EB, Singh R, Lillard JW. Subtype-specific analysis of gene co-expression networks and immune cell profiling reveals high-grade serous ovarian cancer subtype linkage to variable immune microenvironment. J Ovarian Res. 2024;17:240. 39627836 10.1186/s13048-024-01556-4 PMC11613732

[49] Khashaba M, Fawzy M, Abdel-Aziz A, Eladawei G, Nagib R. Subtyping of high-grade serous ovarian carcinoma: histopathological and immunohistochemical approach. J Egypt Natl Canc Inst. 2022;34:6. 35138498 10.1186/s43046-022-00104-9 PMC8827304

[50] Ren Y, Li R, Feng H, Xie J, Gao L, Chu S, Li Y, Meng FL, Ning YS. Single-cell sequencing reveals effects of chemotherapy on the immune landscape and TCR/BCR clonal expansion in a relapsed ovarian cancer patient. Front Immunol. 2022;13:985187. 36248860 10.3389/fimmu.2022.985187 PMC9555851

[51] Xiao Y, Bi M, Guo H, Li M. Multi-omics approaches for biomarker discovery in early ovarian cancer diagnosis. EBioMedicine. 2022;79:104001. 35439677 10.1016/j.ebiom.2022.104001 PMC9035645

[52] Liu X, Xu L, Li J, Yao PY, Wang W, Ismail H, Wang H, Liao B, Yang Z, Ward T, Ruan K, Zhang J, Wu Q, He P, Ding X, Wang D, Fu C, Dou Z, Yan F, Wang W, Liu X, Yao X. Mitotic motor CENP-E cooperates with PRC1 in temporal control of central spindle assembly. J Mol Cell Biol. 2020;12:654–65. 31174204 10.1093/jmcb/mjz051 PMC7683015

[53] Yang Y, Wu G, Sancar A, Hogenesch JB. Mutations of the circadian clock genes Cry, Per, or Bmal1 have different effects on the transcribed and nontranscribed strands of cycling genes. Proc Natl Acad Sci. 2024;121:e2316731121. 38359290 10.1073/pnas.2316731121 PMC10895256

[54] Gallardo A, Belmonte-Reche E, Marti-Marimon M, Domingo-Reinés J, Peris G, López-Onieva L, Fernández-Rengel I, Xie J, Tristán-Ramos P, Bellora N, Sánchez-Pozo A, Estévez AM, Heras SR, Marti-Renom MA, Landeira D. BMAL1-TRIM28 represses transposable elements independently of CLOCK in pluripotent cells. Nat Commun. 2025;16:8250. 40931039 10.1038/s41467-025-63778-4 PMC12423293

[55] Schrader LA, Ronnekleiv-Kelly SM, Hogenesch JB, Bradfield CA, Mc Malecki K. Circadian disruption, clock genes, and metabolic health. J Clin Invest. 2024;134:e170998. 39007272 10.1172/JCI170998 PMC11245155

[56] An Y, Yuan B, Xie P, Gu Y, Liu Z, Wang T, Li Z, Xu Y, Liu Y. Decoupling PER phosphorylation, stability and rhythmic expression from circadian clock function by abolishing PER-CK1 interaction. Nat Commun. 2022;13:3991. 35810166 10.1038/s41467-022-31715-4 PMC9271041

[57] Tamayo AG, Duong HA, Robles MS, Mann M, Weitz CJ. Histone monoubiquitination by Clock-Bmal1 complex marks Per1 and Per2 genes for circadian feedback. Nat Struct Mol Biol. 2015;22:759–66. 26323038 10.1038/nsmb.3076 PMC4600324

[58] Hu Z, Tan H, Ye Y, Xu W, Gao J, Liu L, Zhang L, Jiang J, Tian H, Peng F, Tu Y. NIR-actuated ferroptosis nanomotor for enhanced tumor penetration and therapy. Adv Mater. 2024;36:e2412227. 39370589 10.1002/adma.202412227

[59] Guo YD, Xu YY, Bao QQ, Shen C, Ni DL, Hu P, Shi JL. Endogenous Copper for Nanocatalytic Oxidative Damage and Self-Protection Pathway Breakage of Cancer. ACS Nano. 2021;15(10):16286–97. 34652919 10.1021/acsnano.1c05451

[60] Feng QS, Fang WM, Guo YD, Hu P, Shi JL. Nebulized Therapy of Early Orthotopic Lung Cancer by Iron-Based Nanoparticles: Macrophage-Regulated Ferroptosis of Cancer Stem Cells. J Am Chem Soc. 2023;145(44):24153–65. 37897426 10.1021/jacs.3c08032

[61] Zhu XY, Wang TY, Jia HR, Wu SY, Gao CZ, Li YH, Zhang X, Shan BH, Wu FG. A ferroptosis-reinforced nanocatalyst enhances chemodynamic therapy through dual H_2_O_2_ production and oxidative stress amplification. J Control Release. 2024;367:892–904. 38278369 10.1016/j.jconrel.2024.01.049

[62] Ga L, Xin Y, Liu H, Zhang Q, Ning W, Wang X, Yu Y, Su M, Gao H. ROS-amplifying nanoregulators: precision thioredoxin reductase-targeted disruption of redox homeostasis overcomes tumor redox resistance. J Control Release. 2026;390:114580. 41456804 10.1016/j.jconrel.2025.114580

[63] Xu YY, Guo YD, Chen L, Ni DL, Hu P, Shi JL. Tumor chemical suffocation therapy by dual respiratory inhibitions. Chem Sci. 2021;12(22):7763–9. 34168829 10.1039/d1sc00929j PMC8188586

