# Supplementary material for: Biomimetic fusion nanosystem from ginger exosomes and tumor cell membranes: boosting PLK1-targeted therapy in BRCA-heterogeneous HGSOC
Source: J Nanobiotechnology. 2026 Jun 19;24:819. doi: 10.1186/s12951-026-04656-z (PMC13528168; doi:10.1186/s12951-026-04656-z)
Supplement: Supplementary file 1 [file 12951_2026_4656_MOESM1_ESM.docx]

Supporting information

**Biomimetic fusion nanosystem from ginger exosomes and tumor cell membranes: Boosting PLK1-targeted therapy in BRCA-heterogeneous ovarian cancer**

Xue Zhou^1#^, Yutang Huang^1#^, Chenyi Li^1^, Wenlu Mo^1^, Weifeng Xia^1^, Ruiqin Du^1^, Lanxiang Wu^1*^, Hongbo Zhao^2*^

1 Laboratory of Pharmacogenetics and Pharmacogenomics, College of Pharmacy, Chongqing Medical University, Chongqing 400016, P.R. China.

2 Obstetrics & Gynecology Hospital of Fudan University, Shanghai Key Lab of Reproduction and Development, Shanghai Key Lab of Female Reproductive Endocrine Related Diseases, 200433, Shanghai, China.

^#^These authors contributed equally.

*Corresponding author:

Hongbo Zhao, zhaohongbo1506@fckyy.org.cn;

Lanxiang Wu, lxwu@cqmu.edu.cn

**Table S1** Overview of HGSOC Patients and Samples and of Established Organoid Lines

| Patient/HGSOC Sample No. | HGSOC Type^a^ | Brca mutation type^b^ | HGSOC organoid line^c^ | Passaging Time |
| --- | --- | --- | --- | --- |
| 1 | IVA | - | - |  |
| 2 | IIIC | - | - |  |
| 3 | IIIC | - | HGSOC_O_1 | short-term |
| 4 | IIIC | - | HGSOC_O_2 | Long-term |
| 5 | IVB | Brca 2 | HGSOC_O_3 | Long-term |
| 6 | IIIC | - | HGSOC_O_4 | Long-term |
| 7 | IVB | - | - |  |
| 8 | IVB | - | - |  |
| 9 | IIIC | - | HGSOC_O_5 | short-term |
| 10 | IIIV | Brca 1 | HGSOC_O_6 | Long-term |
| 11 | IIB | - | - |  |
| 12 | IVB | Brca 2 | HGSOC_O_7 | Long-term |
| 13 | IVB | - | - |  |
| 14 | IIIB | Brca 2 | HGSOC_O_8 | short-term |
| 15 | IIIC | - | - |  |
| 16 | IIIC | - | - |  |
| 17 | IIIC | Brca 1 | HGSOC_O_9 | Long-term |
| 18 | IVB | - | - |  |
| 19 | IVB | - | HGSOC_O_10 | Long-term |
| 20 | IVB | - | - |  |
| 21 | IIIC | Brca 1 | HGSOC_O_11 | short-term |
| 22 | IIB | - | HGSOC_O_12 | Long-term |
| 23 | IVA | - | - |  |
| 24 | IVB | Brca 1 | HGSOC_O_13 | Long-term |
| ^a^HGSOC, high-grade serous ovarian cancer.  ^b^-, Brca wild type.  ^c^-, no organoid derivation. | | | | |

**Table S2** Primer sequences for real-time RT-PCR analysis

| Gene | Forward | Reverse |
| --- | --- | --- |
| *PLK1* | TGCCTGACCATTCCACCAAG | ATGTCACTGAGGTGGCAGTC |
| *GAPDH* | GGGGCTCTCCAGAACATC | TGACACGTTGGCAGTGG |

**Figure S1.** IHC staining of tumor tissue. (Scale bar: 50 μm)

**Figure S2.** HTS of 643 small molecular compounds was used to identify the potential anti-HGSOC drugs in different BRCA orgnoid cultures. The average inhibition ratio of each drug was represented by a scattered point. The red color points indicated the drugs with average inhibition rates ≥70%.

**Figure S3.** The inhibitory effect of Bi2536 at different concentrations on the normal HGSOC cell (IOSE-80).

**Figure S4.** Ki-67 in xenograft tumors. Scale bars: 200 μm.

**Figure S5.** ***In vivo* antitumor efficacy of Bi2536 in UWB1.289 CDX model.** (A) Schematic of the in vivo experimental design and dosing regimen for vehicle control and Bi2536 (25 or 50 mg/kg). (B) Body weight changes during treatment. (C) Representative tumor photographs and (D) quantified tumor weights at endpoint. (E) Tumor growth curves across treatment groups (n = 5). (F) Representative H&E staining of xenograft tumors (Scale bars: 200 μm). (G) Serum biochemical markers of liver (ALT, AST) and kidney (BUN, CREA) function.

**Figure S6.** ***In vivo* antitumor efficacy of Bi2536 in KURAMOCHI CDX model.** (A) Schematic of the in vivo experimental design and dosing regimen for vehicle control and Bi2536 (25 or 50 mg/kg). (B) Body weight changes during treatment. (C) Representative tumor photographs and (D) quantified tumor weights at endpoint. (E) Tumor growth curves across treatment groups (n = 5). (F) Representative H&E staining of xenograft tumors (Scale bars: 200 μm). (G) Serum biochemical markers of liver (ALT, AST) and kidney (BUN, CREA) function.

**Figure S7.** (A) Representative TEM image of GDVs. (B) Size distribution of GDVs determined by nanoparticle tracking analysis. (C) The inhibitory effect of GDVs at different concentration on the HGSOC cells at different time scales. (scale bar: 0.2 μm)

**Figure S8.** 10% SDS-PAGE photographs of TMMSN.

**Figure S9.** CLSM images of UWB1.289 and KURAMOCHI cells cultured with HM@TMMSN NPs for 2 h (scale bar: 100 μm)

**Figure S10.** UV absorption spectra (A) and standard curve (B) of Bi2536 at different concentrations.

**Figure S11.** Curves of Mn release from HM@TMMSN@Bi2536 in different conditions that simulated tumor environments.

**Figure S12.** The inhibitory effect of nano-materials with different concentrations in OVCAR3 cells.

**Figure S13.** Fluorescence images of calcein-AM and PI co-labeled OVCAR3, KURAMOCHI, and UWB1.289 cells after PBS and HM@TMMSN@Bi2536 treatment (Scale bar: 100 μm).

**Figure S14.** Immunohistochemical analysis of PLK1 signaling in tumor tissues (total PLK1 and phosphorylated PLK1, Scale bar: 200 μm).

**Figure S15**. Bi2536 Standard Solution (A) and Sample Extraction Ion Chromatogram (B); (C) The concentration of Bi2536 in tumor tissue.

**Figure S16.** Hemolytic analysis of HM@TMMSN and HM@TMMSN@Bi2536.
